# A BRAF-activated noncoding RNA attenuates clear cell renal cell carcinoma *via* repression of glucose-6-phosphate dehydrogenase

**DOI:** 10.1016/j.jbc.2025.108247

**Published:** 2025-01-31

**Authors:** Wenjing Liu, Yueli Ni, Honggang Bai, Xiangjie Liu, Asif Shahzad, Kun Cui, Qiuxin Duan, Ziyuan Bai, Yurong Dong, Zihan Yi, Buqing Sai, Yingmin Kuang, Chen Guo, Yuechun Zhu, Qiao Zhang, Zhe Yang

**Affiliations:** 1 Department of Biochemistry and Molecular Biology, School of Basic Medical Sciences, Kunming Medical University, Yunnan, Kunming, PR China; 2 Department of Clinical Laboratory, The Second Hospital of Jingzhou, Jingzhou, Hubei, PR China; 3 Department of Pathology, The First Affiliated Hospital of Kunming Medical University, Yunnan, Kunming, PR China; 4 Department of Medical Oncology, The Third Affiliated Hospital of Kunming Medical University (Tumor Hospital of Yunnan Province), Yunnan, Kunming, PR China; 5 Department of Organ Transplantation, The First Affiliated Hospital of Kunming Medical University, Yunnan, Kunming, PR China; 6 Greater Bay Biomedical InnoCenter, Shenzhen Bay Laboratory, Shenzhen, PR China

**Keywords:** ccRCC, BANCR, G6PD, dimer formation, glucose metabolic flow

## Abstract

Clear cell renal cell carcinoma (ccRCC) is a disease rooted in metabolic disorders, distinguished by abnormally high activity of glucose 6-phosphate dehydrogenase (G6PD). G6PD serves as a key rate-limiting enzyme in the pentose phosphate pathway. Meanwhile, BRAF-activated noncoding RNA (BANCR) has emerged as a crucial regulatory factor linked to various cancers. The expression pattern of BANCR varies across different cancer types, exhibiting apparent duality in its function. However, the precise role and underlying mechanisms of BANCR in ccRCC tumorigenesis remain incompletely understood. Our study indicated that BANCR was downregulated in ccRCC and influenced cell survival by modulating cell proliferation, apoptosis, and G6PD enzyme activity. The underlying mechanism was that BANCR could directly bind to G6PD through a long noncoding RNA–protein interaction, ultimately inhibiting G6PD activity by impeding its dimer formation. Moreover, BANCR exhibited the capability to modulate the glucose metabolic flow in ccRCC cells. Subsequent experiments demonstrated a significant inhibition of tumor growth *in vivo* upon overexpression of BANCR, and G6PD played a pivotal role in mediating the tumor-suppressive effect of BANCR in ccRCC cells. In conclusion, this study provides novel insights into the molecular pathogenesis of ccRCC, highlights a distinct and new regulatory mechanism responsible for the ectopic overactivation of G6PD in ccRCC progression, and suggests that BANCR-mediated suppression of G6PD activity could emerge as a potential therapeutic strategy for ccRCC treatment.

Renal cell carcinoma (RCC), one of the most prevalent urological cancers worldwide, impacts over 400,000 individuals annually (1). In 2023, the United States alone recorded approximately 81,800 diagnosed cases of renal cancer and renal pelvis cancer, leading to 14,890 deaths (2). Among its subtypes, clear cell renal cell carcinoma (ccRCC) stands out as the predominant histological subtype, constituting 75% of all RCC cases and exhibiting high malignancy (3). Recent data indicates that the incidence and mortality rates of ccRCC are increasing (1, 2). A notable characteristic of ccRCC is its resistance to radiotherapy, chemotherapy, targeted therapy, and immunotherapy, presenting significant treatment challenges. Notably, the average overall survival for patients with pleural metastases is a mere 16 months (4, 5). Although surgical intervention remains the primary treatment approach, the postoperative recurrence rate is still as high as 20% to 50% (6). Given these circumstances, a more profound understanding of the pathogenesis of ccRCC has become a crucial focus in contemporary basic and clinical research.

Recent studies have suggested that ccRCC is fundamentally a metabolic disorder (7, 8). The metabolic reprogramming in this disease involves multiple pathways of sugars, lipids, amino acids, and nucleotides. The literature indicates that ccRCC tumorigenesis is intricately linked to enhanced glycolysis, activation of the pentose phosphate pathway (PPP), inhibition of the tricarboxylic acid (TCA) cycle, and significant accumulation of lipids and glycogen. These accumulations are often removed during standard histological preparations, giving ccRCC its distinct clear cell phenotype (8, 9, 10, 11, 12). However, the primary factors and molecular mechanisms directly linking these metabolic dysregulations to the progression of ccRCC remain inadequately elucidated.

Glucose 6-phosphate dehydrogenase (G6PD) is the rate-limiting enzyme of the PPP and plays a crucial role in maintaining metabolic homeostasis in various cancer cells, including glucose oxidation, lipid synthesis, nucleotide precursor generation, and redox balance (13, 14). Our study revealed abnormally high activity of G6PD in ccRCC, highlighting its significant role in the metabolic reprogramming and tumorigenesis of this cancer type (15, 16, 17). G6PD modulation could occur through the synergistic activation of reactive oxygen species–stimulated NF-κB and pSTAT3 signaling as well as silent information regulator 2 (SIRT2)–induced ubiquitination and SUMO1 modification of the G6PD protein at both transcriptional and post-transcriptional regulatory stages (18, 19). In normal cells, the expression and activity of G6PD are strictly controlled, catalyzing enzyme activity through the formation of dimers or tetramers (20, 21). However, the association between G6PD multimer formation and ccRCC tumorigenesis, as well as its functional implications and regulatory mechanisms in ccRCC progression, still require further investigation. Thus, our research aims to elucidate the regulatory dynamics of G6PD multimers and explore potential therapeutic pathways for G6PD-driven ccRCC through functional and mechanistic evaluations.

Prior research has indicated that long noncoding RNAs (lncRNAs) longer than 200 base pairs (bp) can act as molecular scaffolds to modify the function of some proteins at the post-translational level (22). The BRAF-activated noncoding RNA (BANCR) is located on chromosome 9q21.11, with a length of 693 bp, and was initially identified in melanoma cells containing the BRAF^V600E^ mutation (23). Subsequent research found that BANCR expression is abnormal in various cancers, including bladder cancer, colorectal cancer, endometrial cancer, gastric cancer, and hepatocellular carcinoma (24). Its regulatory direction exhibits tissue specificity, and BANCR acts as either an oncogene or a tumor suppressor gene in different cancers. For instance, BANCR was overexpressed in melanoma and influenced cell migration by upregulating CXCL11 (25). Moreover, BANCR significantly increased and enhanced the resistance of gastric cancer cells to cisplatin by stimulating the extracellular signal–regulated kinase 1/2 pathway (26). Conversely, BANCR was downregulated in lung cancer, bladder cancer, and kidney cancer, exhibiting tumor-inhibitory effects (24). Reports suggested that BANCR expression was diminished in ccRCC and might serve as a novel prognostic biomarker for ccRCC patients (27). However, the specific mechanisms by which BANCR impacts ccRCC tumorigenesis and its complex regulatory dynamics during ccRCC progression remain poorly understood.

In this study, we observed that lncRNA BANCR exhibited a downregulation trend in ccRCC, which modulated cell survival by affecting cell proliferation and apoptosis. We identified G6PD as the target of BANCR, and the potential mechanism involved BANCR binding to G6PD through direct lncRNA–protein interactions, thereby reducing G6PD activity by restraining dimer formation. Moreover, BANCR overexpression (OE) regulated glucose metabolism flow within ccRCC cells and inhibited the growth of ccRCC cells *in vivo*. Thus, our work highlighted the significant role of BANCR-mediated inhibition of G6PD activity in regulating ccRCC tumorigenesis and suggested its potential as a therapeutic target for ccRCC.

## Results

### LncRNA BANCR was downregulated in ccRCC

**Figure 1:**
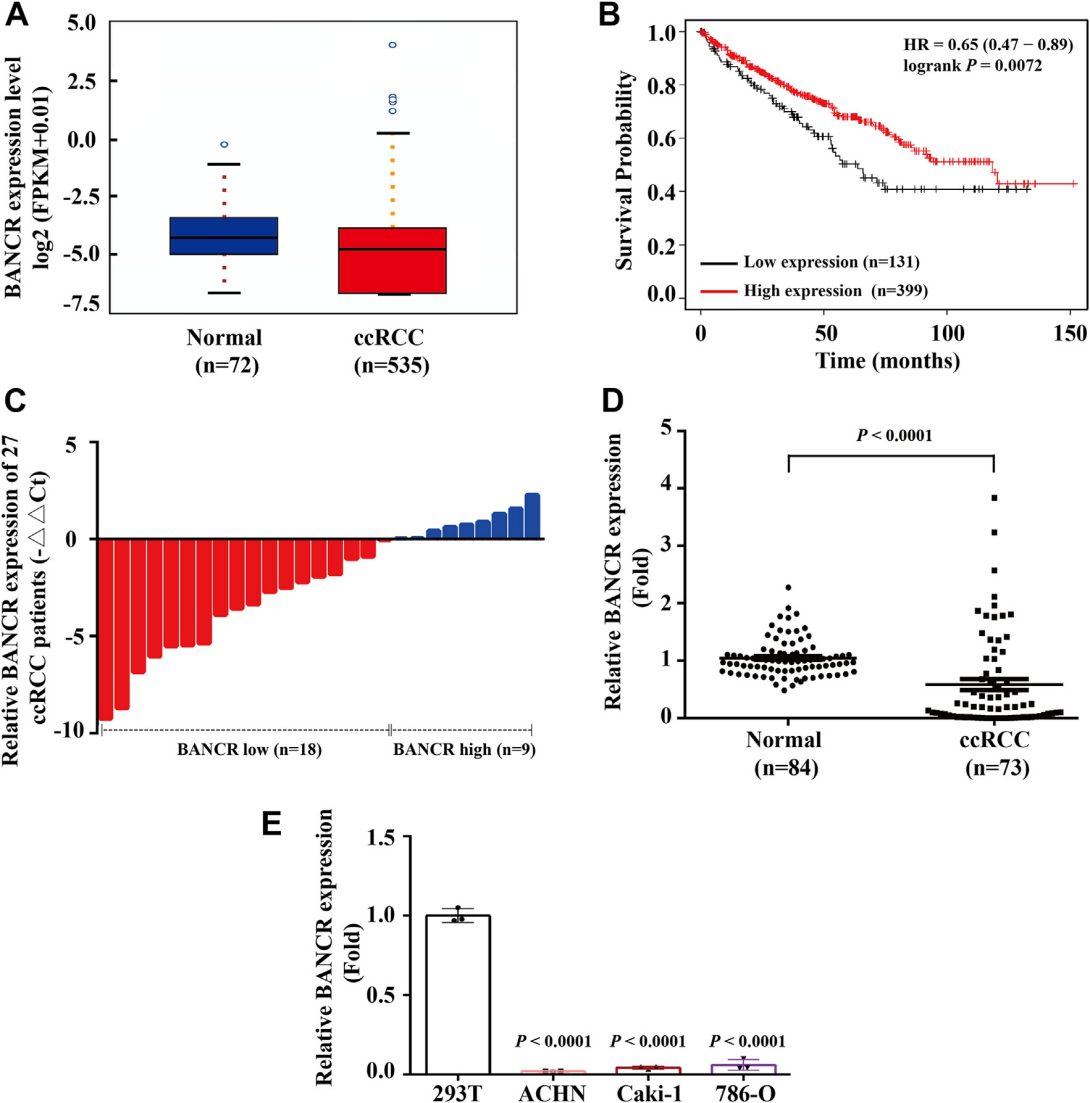
**LncRNA BANCR was downregulated in ccRCC.***A*, the expression profile of BANCR was analyzed by downloading the transcriptome data of ccRCC samples (n = 535) and normal renal tissues (n = 72) from The Cancer Genome Atlas (TCGA) database. Comparison with normal tissues revealed significant differential expression, with *p* = 0.0390 (Mann–Whitney *U* test). *B*, overall survival rates between ccRCC patients with low and high levels of BANCR expression were analyzed by mining the TCGA dataset and integrating it with Kaplan–Meier analyses. *C*, the relative expression levels of BANCR in 27 paired ccRCC samples and their relevant adjacent normal samples were detected using real-time RT–PCR analyses (paired Student's *t* test). *D*, the relative expression levels of BANCR in 84 normal kidney tissues and 73 ccRCC specimens were determined using real-time RT–PCR analyses (unpaired Student's *t* test). *E*, the relative expression levels of BANCR in 293T and ccRCC cell lines (ACHN, Caki-1, and 786-O) were determined using real-time RT–PCR analyses (one-way ANOVA). To standardize real-time RT–PCR analyses, U6 was used as a reference control. Each analysis was performed at least three times. All data were presented as the mean ± SD of three independent experiments. BANCR, BRAF-activated noncoding RNA; ccRCC, clear cell renal cell carcinoma; lncRNA, long noncoding RNA.

To further investigate the expression of BANCR in ccRCC, we utilized The Cancer Genome Atlas (TCGA) dataset for a comprehensive analysis. The results revealed that the presence of BANCR mRNA in ccRCC samples was notably reduced compared with that in normal tissues (Fig. 1*A*). Based on the median expression level of BANCR mRNA, we categorized 530 ccRCC specimens into a BANCR-low group (n = 131) and a BANCR-high group (n = 399). Survival data, stratified according to these groupings, were visualized using Kaplan–Meier plots. The analysis indicated that patients in the BANCR-high group exhibited extended survival durations (Fig. 1*B*).

Subsequently, real-time RT–PCR analysis was conducted to assess the expression of BANCR in 27 pairs of ccRCC tissues and their corresponding adjacent tissues. The results demonstrated that the expression of BANCR in most ccRCC tissues (n = 18) was greatly lower than in the matched adjacent tissues (Fig. 1*C*). Approximately one-third of ccRCC cases exhibit high BANCR expression, further underscoring the heterogeneity and complexity of this disease's pathogenesis (28). In addition, we measured BANCR expression levels in 84 normal kidney tissues and 73 ccRCC samples, observing a significant decrease in BANCR expression in ccRCC samples relative to normal tissues (Fig. 1*D*).

To assess the biological effects of BANCR, we analyzed its expression levels in various RCC cell lines. Real-time RT–PCR results showed that compared with the normal human renal epithelial cell line 293T, the expression of BANCR was markedly reduced in RCC cell lines (including ACHN, 786-O, and Caki-1) (Fig. 1*E*). This finding suggested that BANCR might play a pivotal role in the malignant progression of ccRCC.

### BANCR modulated ccRCC cell survival by affecting cell proliferation and apoptosis

**Figure 2:**
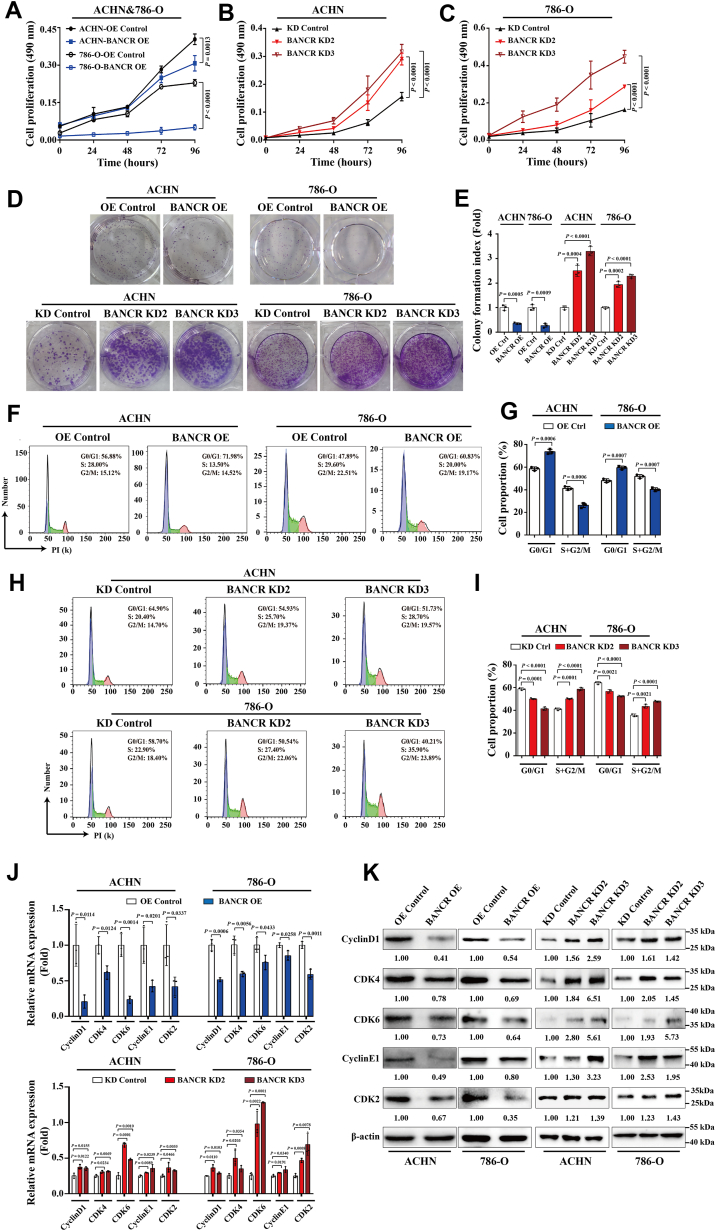
**BANCR OE inhibited ccRCC cell proliferation and changed cell cycle dynamics.***A*–*E*, proliferation rates of stable BANCR–overexpressing cells, BANCR knockdown cells, and relevant control cells were determined by MTS analyses (*A*–*C*), colony-formation analyses (*D*), and quantification assessments (*E*). *F–I,* cell cycle distributions of stable BANCR–overexpressing cells, BANCR knockdown cells, and relevant control cells were analyzed by flow cytometry analyses. *J* and *K*, cell cycle–related factors, including CyclinD1, CDK4, CDK6, Cyclin E1, and CDK2, in stable BANCR–overexpressing cells, BANCR knockdown cells, and relevant control cells were detected by real-time RT–PCR analyses (*J*) and Western blot analyses (*K*). U6 was employed as a standardized control in real-time RT–PCR analyses. β-actin was used as a loading control in Western blot assays. All experimental analyses were conducted at least three times. The data were represented as mean ± SD of three independent experiments. *A*–*C*, mixed ANOVAs were used, whereas unpaired Student's *t* tests were used for other analyses. BANCR, BRAF-activated noncoding RNA; ccRCC, clear cell renal cell carcinoma; Ctrl, control; KD, knockdown; MTS, 3-(4,5-dimethylthiazol-2-yl)-5-(3-carboxymethoxyphenyl)-2-(4-sulfophenyl)-2H-tetrazolium; OE, overexpression.

To clarify the effect of BANCR on ccRCC tumorigenesis, we employed a lentiviral vector carrying BANCR OE sequences to induce OE of BANCR in ACHN and 786-O cells. In addition, we employed three shRNA-loaded lentiviral vectors to knock down BANCR expression. Through mRNA level analysis, we confirmed the effectiveness of these interventions and successfully established cell lines with BANCR OE and knockdown (Fig. S1). In these modified cell lines, we observed a significant decrease in the growth rate of BANCR-overexpressing cells compared with their respective control cells, whereas BANCR knockdown cells exhibited an increased growth rate (Fig. 2, *A*–*C*). This result suggests that BANCR plays a crucial role in modulating the proliferation of ccRCC cells. Subsequent colony-formation experiments further confirmed that OE of BANCR notably weakened the colony-forming ability of ACHN and 786-O cells, whereas cells exhibited enhanced colony formation following BANCR knockdown (Fig. 2, *D* and *E*).

To gain deeper insights into the specific effects of BANCR on ccRCC cell proliferation, we conducted cell cycle distribution measurements. The analysis results indicated that compared with the control group, the G0/G1 phase ratio of ACHN and 786-O cells overexpressing BANCR significantly increased (Fig. 2, *F* and *G*). Conversely, the G0/G1 phase ratio of ACHN and 786-O cells with BANCR knockdown dramatically diminished (Fig. 2, *H* and *I*). As expected, cell cycle regulatory factors such as CyclinD1, CDK4, CDK6, CyclinE1, and CDK2, which are known to play a pivotal role in the pathogenesis of ccRCC (6, 16), were greatly reduced at both mRNA and protein levels following BANCR OE in ACHN and 786-O cells. In contrast, knockdown of BANCR in ACHN and 786-O cells resulted in significant upregulation of these cell cycle–related regulatory factors (Fig. 2, *J* and *K*). These data collectively suggested that OE of BANCR inhibited the proliferation of ccRCC cells by downregulating cell cycle proteins.

**Figure 3:**
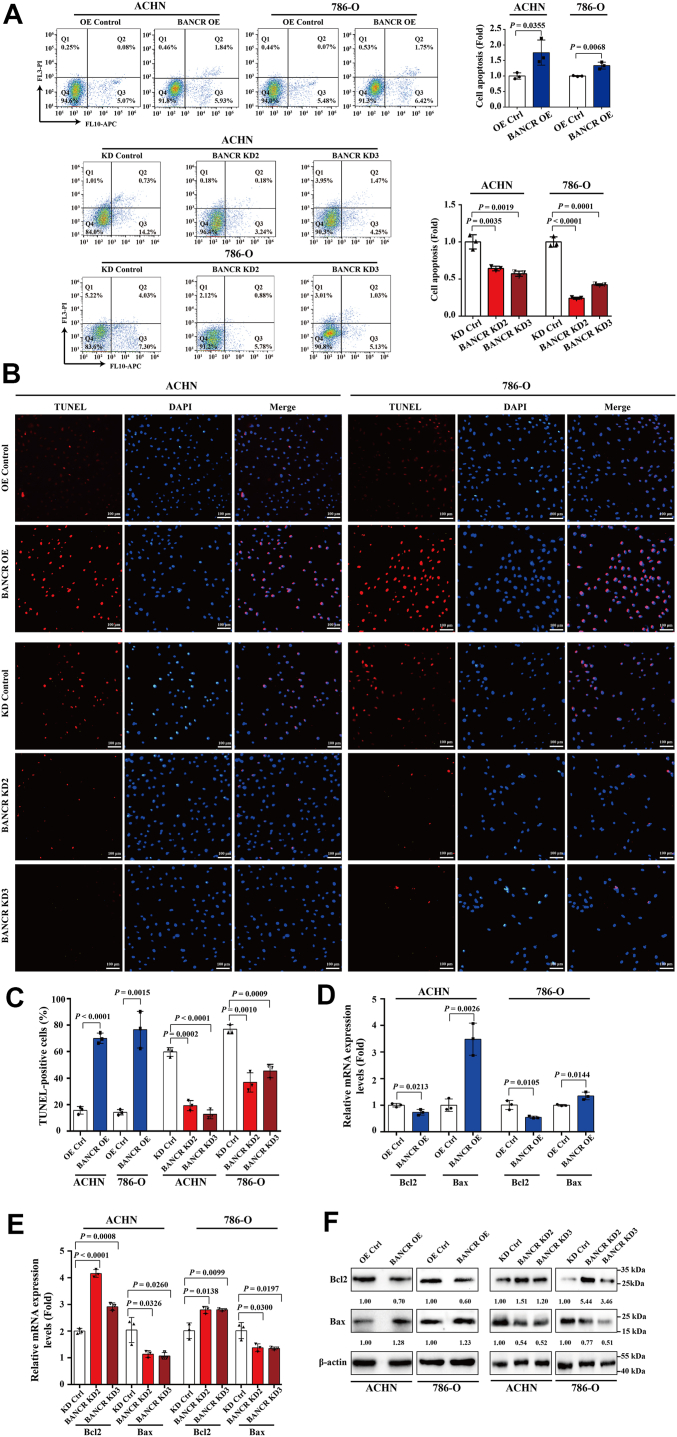
**BANCR overexpression promoted ccRCC cell apoptosis.***A*–*C*, cell apoptosis was measured by flow cytometry (*A*) and the TUNEL assays (*B* and *C*) in stable BANCR–overexpressing cells, BANCR knockdown cells, and relevant control cells. *D*–*F*, apoptosis-related factors Bax and Bcl-2 in stable BANCR–overexpressing cells, BANCR knockdown cells, and relevant control cells were detected by real-time RT–PCR (*D* and *E*) and Western blot analyses (*F*). U6 was employed as a standardized control in real-time RT–PCR analyses. β-actin was used as a loading control in Western blot analyses. All experimental analyses were performed at least three times. The data were shown as mean ± SD of three independent experiments. Statistical analyses were conducted using unpaired Student's *t* tests. BANCR, BRAF-activated noncoding RNA; ccRCC, clear cell renal cell carcinoma.

Having observed that BANCR silencing could enhance the survival rate of ccRCC cells, we further explored the effect of BANCR on the apoptosis of ccRCC cells. Through flow cytometry and double-staining analysis, we found that the apoptosis rate of BANCR-overexpressing cells was notably increased compared with control cells, whereas the opposite trend was observed in BANCR knockdown cells (Fig. 3*A*). In addition, TUNEL detection results indicated that the proportion of TUNEL-positive cells increased after overexpressing BANCR, whereas it decreased following suppression (Fig. 3, *B*–*D*). Further real-time RT–PCR and Western blot analysis indicated that OE of BANCR greatly downregulated the level of the antiapoptotic protein Bcl2 in ACHN and 786-O cells, whereas the proapoptotic protein Bax was upregulated at both mRNA and protein levels (Fig. 3, *E* and *F*). In contrast, these expression patterns were reversed in ACHN and 786-O cells upon BANCR knockdown. These data clearly demonstrated that BANCR had the potential to promote apoptosis in ccRCC cells by modulating the expression of apoptosis-associated proteins Bcl2 and Bax. In summary, the compiled data suggested that BANCR modulated the survival rate of ccRCC cells through its impacts on cell proliferation and apoptosis, providing new potential targets for the treatment of ccRCC.

### BANCR bound with G6PD through direct lncRNA–protein interaction

Considering that the downregulation of BANCR expression seems to have carcinogenic effects in ccRCC, we proceeded to explore the potential underlying mechanisms. The existing literature suggests that post-translational modifications of metabolic enzymes in lncRNA-mediated energy metabolism pathways play a crucial role in the occurrence and progression of tumors (22). However, the specific role of BANCR in modulating metabolic reprogramming of cancer cells remains unclear. Based on previous research, G6PD serves as a central mediator for cancer cell metabolic reprogramming and ccRCC tumorigenesis (6, 13, 14, 16, 18, 19). Our study focused on exploring whether BANCR may interact with G6PD and influence its enzyme activity. The activity of G6PD depends on the binding of the cofactor NADP^+^ and the formation of dimer or tetramer structures. G6PD possesses a "catalytic" NADP^+^ site and a "structural" NADP^+^ site, where multiple severe mutations associated with G6PD deficiency are located. These key mutations (such as V213L to G447R, G488S, or G488V) are situated near the dimer surface and physically interact with the "structural" NADP^+^, which is crucial for maintaining the stability and integrity of active G6PD (21, 29, 30, 31).

**Figure 4:**
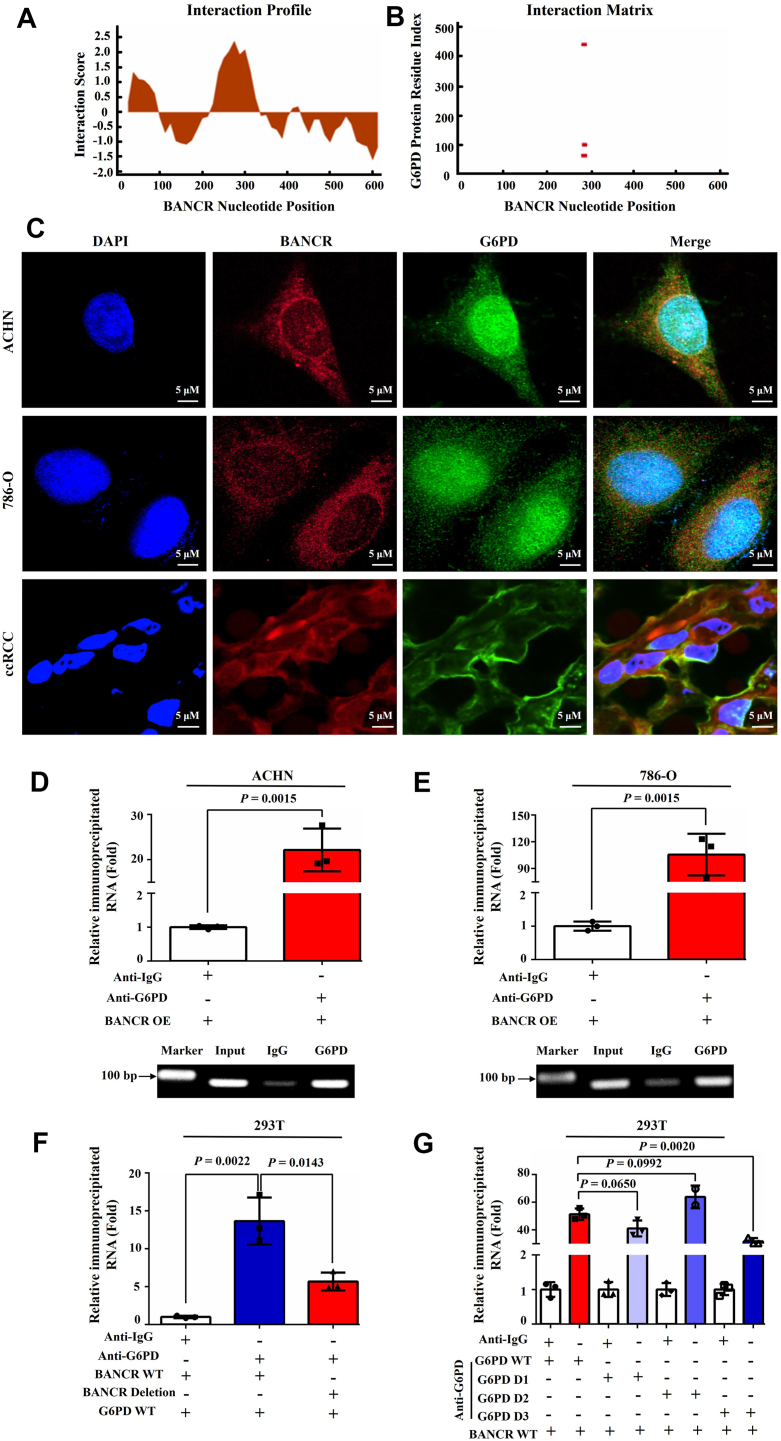
**BANCR bound to G6PD through direct RNA–protein interaction.***A* and *B*, potential RNA–protein interactions between BANCR nucleotides and G6PD amino acids were predicted by the *cat*RAPID software platform. *C*, the expression and colocalization of BANCR and G6PD in ccRCC cell lines (*top* two panels) and ccRCC tumor specimens (*bottom panel*) were analyzed by FISH assays. *D* and *E*, the interaction between BANCR and G6PD was investigated by R-IP analyses using G6PD antibodies or IgG as negative controls in ACHN (*D*) and 786-O (*E*) cells overexpressing BANCR. The relevant analyses included real-time PCR (as shown in the figure above) and PCR (as shown in the figure below). *F*, R-IP assays were conducted in 293T cells transfected with G6PD overexpression plasmid and BANCR wildtype or BANCR-G6PD binding site deletion plasmid. *G*, R-IP assays were performed in 293T cells transfected with BANCR overexpression plasmid and G6PD wildtype or G6PD-BANCR binding site deletion plasmid. All experimental analyses were performed at least three times. The data were shown as mean ± SD from three independent experiments. Statistical analyses were conducted using unpaired Student's *t* tests. BANCR, BRAF-activated noncoding RNA; G6PD, glucose 6-phosphate dehydrogenase; R-IP, RNA immunoprecipitation.

To investigate whether BANCR can directly bind to the G6PD protein and alter its activity, we first used bioinformatics methods and the *cat*RAPID software platform to predict potential interaction sites between BANCR nucleotides and G6PD amino acids. The analysis results showed that BANCR at 276 to 327 nt has a high tendency to bind to target molecules (Fig. 4*A*) and may interact with the 51 to 102, 90 to 141, and 426 to 477 amino acid regions of G6PD (Fig. 4*B*), with the 90 to 141 region being the most probable, followed by the 426 to 477 and 51 to 102 regions. The effectiveness of this prediction was further validated through RNA–Protein website prediction and molecular docking analysis between BANCER and G6PD (Fig. S2). To validate these predictions, we performed RNA FISH testing in ccRCC cell lines and human ccRCC tumor tissues to examine the interaction between BANCR and G6PD. As demonstrated in Figure 4*C*, BANCR and G6PD were mainly coexpressed and colocalized in the cytoplasm of cell lines and ccRCC tumor cells, which not only confirmed the ectopic expression of G6PD in ccRCC but also suggested a potential interaction between BANCR and G6PD (6, 15, 16, 17, 18, 19).

Subsequently, we used real-time PCR (Fig. 4, *D* and *E*, *top*) and agarose gel electrophoresis PCR (Fig. 4, *D* and *E*, *bottom*) for RNA immunoprecipitation (R-IP) assay to verify the BANCR–G6PD interaction. The data confirmed that compared with the IgG control group, BANCR was notably immunoprecipitated by the G6PD protein (Fig. 4, *D* and *E*), indicating that BANCR directly binds to G6PD *in vivo*. To further validate whether nucleotides 276 to 327 of BANCR were the primary binding region for G6PD, we used immunoprecipitation of the BANCR–G6PD complex from 293T cells transfected with a G6PD OE plasmid and BANCR wildtype or binding site (276–327) deletion plasmid. The R-IP assay findings revealed that compared with the BANCR wildtype group, the association rate of BANCR–G6PD in the BANCR binding site deletion group was markedly decreased to roughly 41.6% (Fig. 4*F*), indicating the essential role of nucleotides 276 to 327 of BANCR in its interaction with G6PD. Furthermore, to determine the binding site where G6PD is most likely to interact with BANCR, we performed another R-IP assay in 293T cells transfected with a BANCR OE plasmid and G6PD wildtype or binding site deletion plasmid. As depicted in Figure 4*G*, G6PD lacking the amino acid region 426 to 477 (G6PD D3) exhibited dramatically reduced interaction with BANCR. In comparison, the binding affinities between BANCR and G6PD with deletions in regions 51 to 102 (G6PD D1) or 90 to 141 (G6PD D2) remained relatively unaffected. Taken together, our results suggested that BANCR directly associated with G6PD, potentially influencing the activity of G6PD through this lncRNA–protein interaction.

### BANCR downregulated G6PD activity through restraining its dimer formation

**Figure 5:**
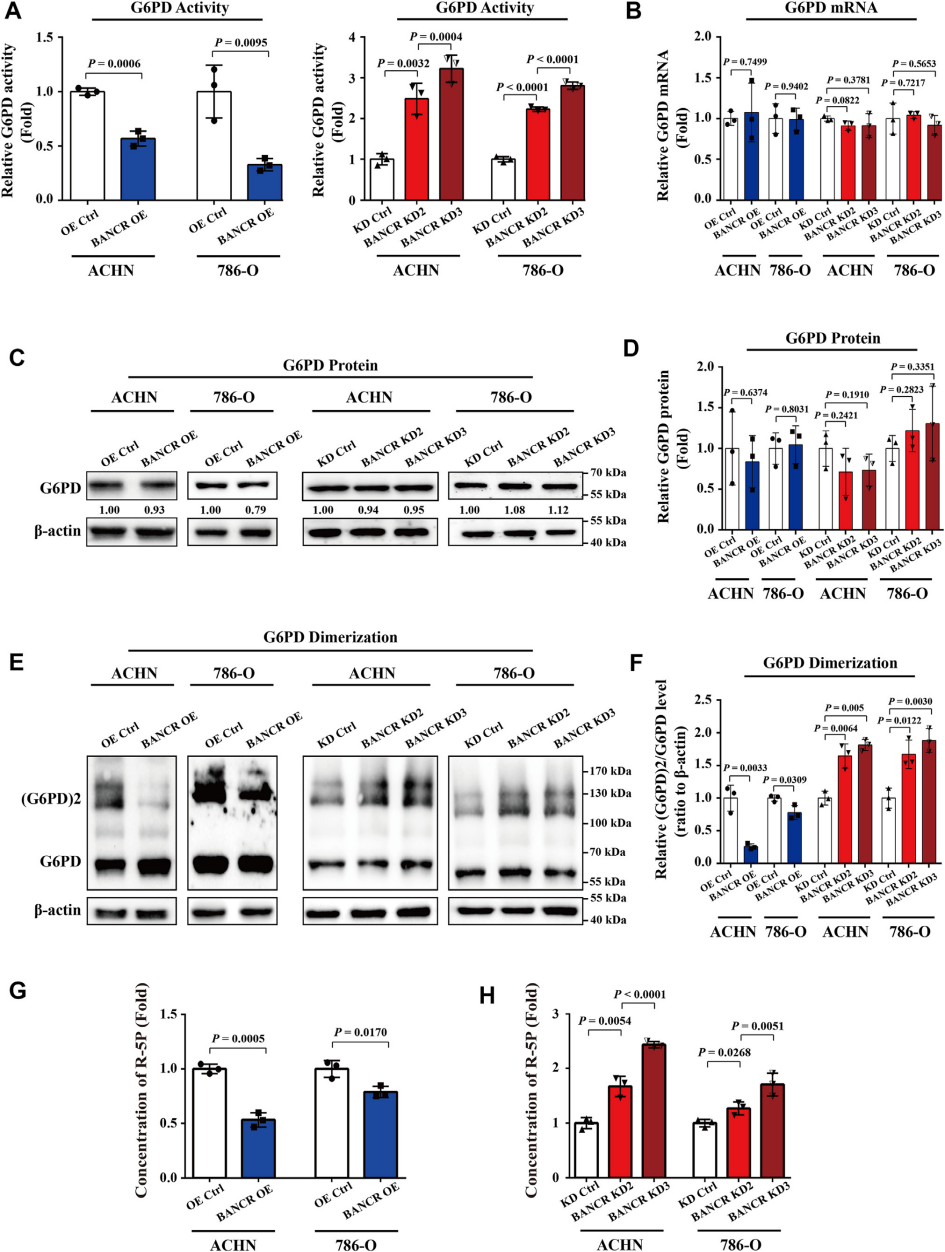
**BANCR restrained G6PD activity by inhibiting its dimer formation.***A*, the relative G6PD activities in stable BANCR–overexpressing cells, BANCR knockdown cells, and relevant control cells were analyzed by the G6PD enzyme activity assay kits. *B*–*D*, the mRNA and protein expression levels of G6PD in stable BANCR–overexpressing cells, BANCR knockdown cells, and relevant control cells were detected using real-time RT–PCR analyses (*B*) and Western blot analyses (*C* and *D*). *E* and *F*, relative dimer and monomer expression levels of G6PD in stable BANCR–overexpressing cells, BANCR knockdown cells, and relevant control cells were analyzed using glutaraldehyde crosslinked Western blot analyses. Representative Western blot images (*C* and *E*) and quantitative analysis statistical data (*D* and *F*) were presented. *G* and *H*, the relative concentration of ribose-5-phosphate in stable BANCR–overexpressing cells, BANCR knockdown cells, and relevant control cells were analyzed using the R-5P ELISA kits. U6 was employed as a standardized control in real-time RT–PCR analyses. β-actin was used as a loading control in Western blot analyses. All experimental analyses were performed at least three times. The data were shown as mean ± SD from three independent experiments. Statistical analyses were conducted using unpaired Student's *t* tests. BANCR, BRAF-activated noncoding RNA; G6PD, glucose 6-phosphate dehydrogenase.

The results suggested that BANCR may facilitate ccRCC tumorigenesis by interacting with the key metabolic regulator G6PD. Our laboratory's previous research has found that the formation of G6PD dimer is crucial for its catalytic enzyme activity and closely related to the progression of ccRCC (19). Therefore, we explored whether BANCR can suppress G6PD activity by inhibiting dimer formation. In the initial stage, we evaluated G6PD activity, and its expression changes at the mRNA and protein levels in ccRCC cells with OE or knockdown of BANCR. As depicted in Figure 5*A*, with the increase of BANCR expression, G6PD activity decreased, whereas in cells with BANCR inhibition, G6PD activity significantly increased. However, through real-time RT–PCR and Western blot analysis, we observed that the expression levels of G6PD, both mRNA and protein, did not show significant changes in ccRCC after overexpressing or silencing BANCR (Fig. 5, *B*–*D*). This led us to speculate that BANCR may regulate its activity by altering the formation of G6PD homodimers in ccRCC. To verify this speculation, we used glutaraldehyde crosslinking assay to evaluate the impact of BANCR on G6PD monomers and dimers. Subsequent Western blot results showed a significant decrease in the ratio of dimeric G6PD to monomeric G6PD in cells with increased BANCR expression. In contrast, after knocking down BANCR in ACHN and 786-O cells, the formation and corresponding proportion of G6PD dimer exhibited an opposite increasing trend (Fig. 5, *E* and *F*), indicating that the rate of the PPP was enhanced. This hypothesis was further confirmed in the quantitative analysis of ribose-5-phosphate (R-5P) concentration in BANCR-overexpressing and knockdown cells (Fig. 5, *G* and *H*), which showed that BANCR OE greatly attenuated R-5P levels by inhibiting G6PD dimer formation.

**Figure 6:**
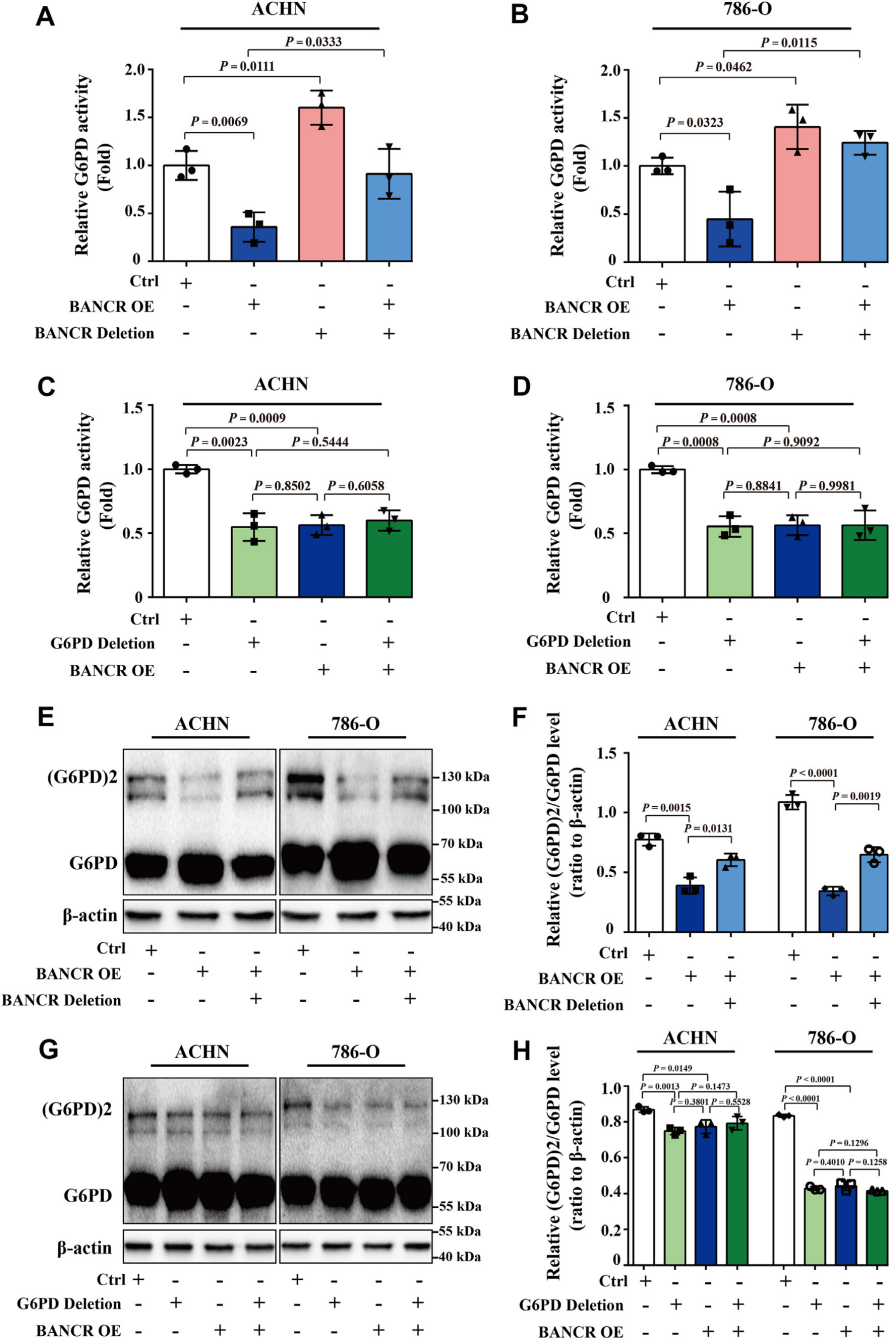
**BANCR or G6PD binding site mutation attenuated the effects of BANCR on G6PD activity and dimer formation.***A* and *B*, the relative G6PD activities in ACHN (*A*) or 786-O (*B*) cells transfected with BANCR OE and/or BANCR-G6PD binding site deletion plasmids were analyzed by the G6PD enzyme activity assay kits. *C* and *D*, the relative G6PD activities of ACHN (*C*) or 786-O (*D*) cells transfected with BANCR OE and/or G6PD-BANCR binding site deletion plasmids were analyzed by the G6PD enzyme activity assay kits. *E*–*H*, the relative dimer and monomer expression levels of G6PD in ACHN-BANCR OE, ACHN-BANCR Deletion (BANCR with G6PD binding sites deletion) (*E* and *F*), 786-O-BANCR OE, 786-O-G6PD Deletion (G6PD with BANCR binding sites deletion) (*G* and *H*), and relevant control cells were analyzed using glutaraldehyde crosslinked Western blot analyses. Representative Western blot images (*E* and *G*) and quantitative analysis statistical data (*F* and *H*) were presented. β-actin was used as a loading control in Western blot analyses. All experimental analyses were performed at least three times. The data were shown as mean ± SD from three independent experiments. Statistical analyses were conducted using unpaired Student's *t* tests. BANCR, BRAF-activated noncoding RNA; G6PD, glucose 6-phosphate dehydrogenase; OE, overexpression.

Furthermore, to investigate the influence of the lncRNA–protein interaction between BANCR and G6PD, we analyzed the activity of G6PD in ccRCC cells lacking BANCR or G6PD binding regions. As illustrated in Figure 6, *A* and *B*, deletion of the binding region of BANCR in ACHN and 786-O cells significantly increased and restored G6PD activity compared with the control and BANCR-overexpressing group, respectively. Simultaneously, as shown in Figure 6, *C* and *D*, compared with the control group, the enzyme activity of G6PD (the second and the third columns) was notably reduced, highlighting the importance of the G6PD binding sites (426–477 amino acid region) in G6PD activity, and BANCR can indeed markedly diminish the enzyme activity of G6PD in its natural state. Comparing the second column with the fourth column, it was found that the ratio was almost negligible, suggesting that even overexpressed BANCR cannot interact with G6PD or influence its enzymatic activity if the G6PD binding site was removed. This further underscored the importance of the interaction between the G6PD binding site and BANCR. Finally, comparing the third column with the fourth column revealed that in the case of OE of BANCR, if the G6PD binding site was removed, BANCR cannot bind to G6PD. In this scenario, G6PD activity may be upregulated following transfection with a G6PD deletion plasmid, but because of the crucial role of this site in G6PD activity, the mutant competed with the wildtype, leading to a decrease in G6PD activity, resulting in no significant variation between these two groups or columns.

These findings indicate that lncRNA–protein interactions play a critical role in BANCR-mediated G6PD activity. However, further research is needed to determine whether the absence of binding sites for BANCR or G6PD reduces its activity by inhibiting the dimerization of G6PD in ccRCC. Therefore, we used a glutaraldehyde crosslinking assay to investigate the effect of BANCR or G6PD binding site deletion on the formation of G6PD monomers and dimers. The results showed that the ratio of dimeric G6PD to monomeric G6PD was greatly reduced in ACHN or 786-O cells overexpressing BANCR. When BANCR deletion transfection was performed, this reduction was reversed (Fig. 6, *E* and *F*). Similarly, the formation of G6PD dimers and the change in the ratio of dimeric G6PD to monomeric G6PD were consistent with the pattern of changes in G6PD activity when the G6PD binding site was absent (Fig. 6, *G* and *H*). Overall, these findings suggested that BANCR might inhibit G6PD activity by suppressing the formation of G6PD active dimers in ccRCC.

### BANCR mediated glucose metabolism in ccRCC cells

**Figure 7:**
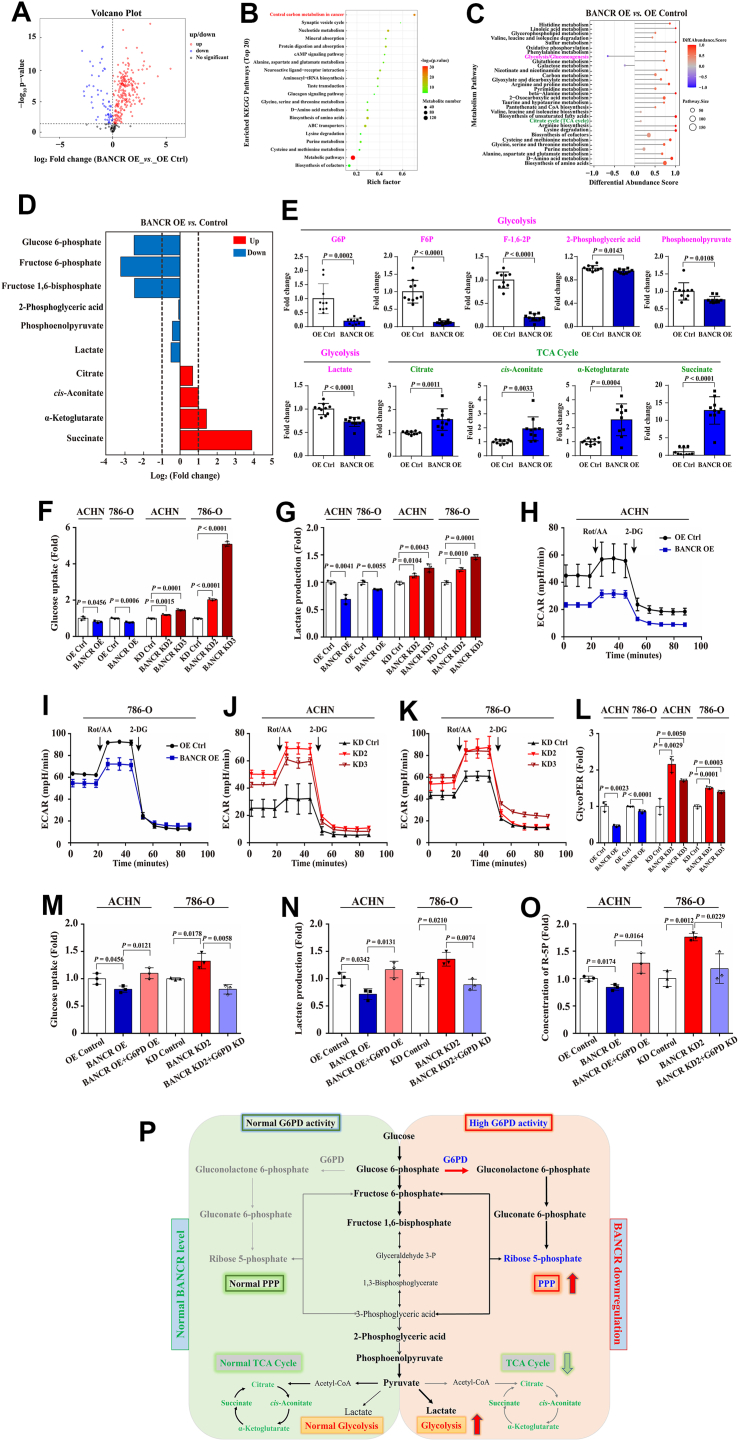
**BANCR mediated the glucose metabolism in ccRCC cells.***A*–*C*, targeted glucose metabolomics analyses were conducted in ACHN-BANCR OE and relevant control cells (n = 10). The volcano plot (*A*) showing the distribution of differential metabolites, the top 20 enriched KEGG pathways (*B*), and the differential abundance score plot of all differential metabolic pathways (*C*) are shown. *D* and *E*, significant differences in glycolysis and TCA cycle–related metabolites were analyzed. *F* and *G*, the glucose uptake (*F*) and lactate production (*G*) in BANCR-overexpressing cells, BANCR knockdown cells, and relevant control cells were analyzed using glucose and lactate concentration detection kits. *H*–*L*, ECAR (*H–K*) and glycoPER (*L*) in stable BANCR–overexpressing cells, BANCR knockdown cells, and relevant control cells were analyzed by the Seahorse XFe 24 analyzer. *M–O*, the glucose uptake (*M*), lactate production (*N*), and ribose-5-phosphate (*O*) concentration in ACHN-BANCR OE, ACHN-BANCR OE+G6PD OE, 786-O-BANCR KD2, 786-O-BANCR KD2+G6PD KD, and relevant control cells were analyzed using glucose, lactate, and ribose-5-phosphate concentration detection kits, respectively. *P*, the glucose metabolomics of normal BANCR expression in renal cells (*left image*) and downregulated BANCR in ccRCC cells (*right image*) were shown. All analyses for *F*–*O* were performed at least three times. The data were shown as mean ± SD of three independent experiments. Statistical analyses were conducted using unpaired Student's *t* tests. BANCR, BRAF-activated noncoding RNA; ccRCC, clear cell renal cell carcinoma; ECAR, extracellular acidification rate; glycoPER, glycolytic proton efflux rate; G6PD, glucose 6-phosphate dehydrogenase; KEGG, Kyoto Encyclopedia of Genes and Genomes; OE, overexpression; TCA, tricarboxylic acid.

Our previous research has confirmed that G6PD activity is abnormally elevated in ccRCC (16, 19), suggesting that its upstream regulator, BANCR, may play a pivotal role in glucose metabolism reprogramming. To test this hypothesis, we conducted high-throughput metabolomic analysis using UHPLC-QTRAP MS technology in cultured ACHN-BANCR OE and control cells. This analysis covered 623 metabolites, including sugars, lipids, amino acids, nucleotides, vitamins, etc (Fig. S3). The results revealed significant differences in 300 metabolites (Fig. 7*A*). By analyzing the enriched Kyoto Encyclopedia of Genes and Genomes pathway top 20, we found that the central carbon metabolism pathway in cancer showed the most notable changes (Fig. 7*B*). Further examination of the abundance fractions of various metabolism pathways indicated that in the BANCR OE group, the abundance of glycolytic/gluconeogenic metabolites decreased markedly, whereas the abundance of metabolites in the citric acid cycle (TCA cycle) increased greatly (Fig. 7*C*). Compared with the control group, ACHN-BANCR-overexpressing cells exhibited markedly reduced levels of metabolites, such as glucose 6-phosphate, fructose 6-phosphate, fructose 1,6-bisphosphate, 2-phosphoglyceric acid, phosphoenolpyruvate, and lactate in the glycolysis metabolism pathway. Conversely, they showed significantly increased levels of metabolites like citrate, *cis*-aconitate, α-ketoglutarate, and succinate in the TCA cycle (Fig. 7, *D* and *E*).

To further validate the impact of BANCR on the glycolysis rate in stably transfected ccRCC cells, we conducted additional experiments using metabolite detection kits and a Seahorse XF24 analyzer. The results revealed that BANCR OE dramatically reduced glucose uptake and lactate production in ACHN and 786-O cells, whereas the BANCR knockdown group exhibited the opposite trend. In addition, through extracellular acidification rate (ECAR) and glycolytic proton efflux rate (glycoPER) measurements (Fig. 7, *H–L*), we observed that BANCR OE notably reduced the glycolytic activity of BANCR-overexpressing cells, signifying a reduction in aerobic glycolysis. Conversely, compared with control cells, the glycolytic phenotype of BANCR knockdown cells was markedly enhanced, suggesting that BANCR plays a crucial role in mediating glucose metabolism in ccRCC cells. However, the precise mechanism underlying the role of G6PD in BANCR-mediated acceleration of ccRCC glycolysis remains elusive and warrants further investigation.

To delve deeper into this phenomenon, we compared glucose uptake, lactate production, and R-5P content in ACHN-BANCR OE, 786-O-BANCR KD2, and their respective control cells. The results revealed that BANCR OE significantly reduced glucose uptake, lactate production, and R-5P levels in ACHN cells. In contrast, G6PD OE significantly rescued the downregulation of glycolysis and the PPP in ACHN-BANCR OE cells. Similarly, while BANCR knockdown accelerated glycolysis and PPP processes in 786-O cells, G6PD knockdown greatly reversed this effect (Fig. 7, *E* and *F*). These findings strongly underscored the importance of enhanced glycolytic activity when G6PD was downregulated by BANCR in ccRCC. In summary, our comprehensive data strongly supported the notion that BANCR downregulation shifted glucose metabolism in ccRCC cells toward aerobic glycolysis and the PPP, rather than the TCA cycle. This shift was likely achieved through interactions with the downstream target G6PD (Fig. 7*P*).

### BANCR OE limited tumor growth of ccRCC cells *in vivo*

**Figure 8:**
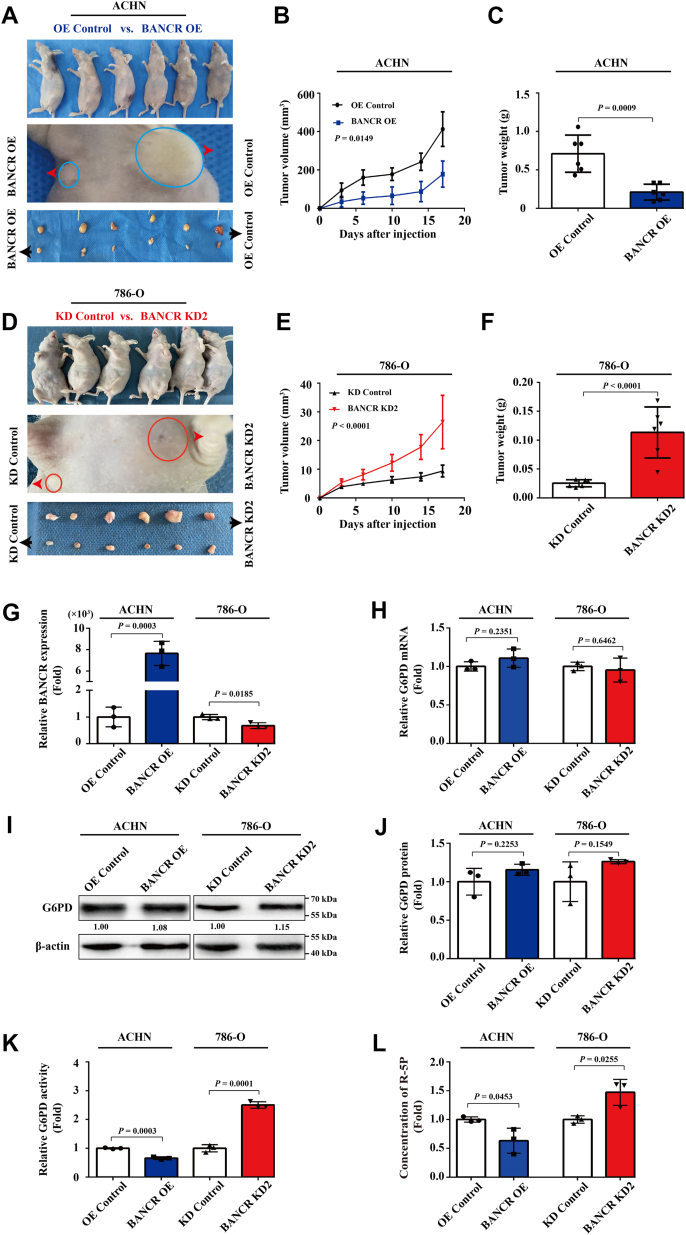
**BANCR OE limited ccRCC tumor growth *in vivo*.***A*–*F*, 1 × 10^7^ ACHN-BANCR OE (*A*–*C*), 786-O-BANCR KD2 (*D–F*), or relevant control cells were subcutaneously injected into nude mice. Images of tumor-bearing mice and their isolated tumors from each group were shown (*A* and *D*). Tumor volumes were monitored and calculated after cell injection (*B* and *E*). Tumor weights were measured after euthanizing the mice (*C* and *F*). *G* and *H*, the relative expression levels of BANCR (*G*) and G6PD (*H*) in each group of tumors were detected using real-time RT–PCR analyses. *I* and *J*, the expression levels of G6PD protein in each group of tumors were analyzed by Western blot analyses (*I*) and statistical analyses (*J*). *K*, the relative G6PD activities of each group of tumors were analyzed by G6PD enzyme activity assay kits. *L*, the relative concentrations of ribose-5-phosphate in each group of tumors were analyzed by R-5P ELISA kits. U6 was employed as a standardized control in real-time RT–PCR analyses. β-actin was used as a loading control for Western blot analyses. *G*–*L* analyses were performed at least three times (n = 3 per group). The data were shown as mean ± SD of three independent experiments. *B* and *E*, mixed ANOVAs were used, whereas unpaired Student's *t* tests were used for other analyses. BANCR, BRAF-activated noncoding RNA; ccRCC, clear cell renal cell carcinoma; G6PD, glucose 6-phosphate dehydrogenase; OE, overexpression.

To further validate the effect of BANCR on the *in vivo* growth of ccRCC cells, we conducted subcutaneous tumor assays. Equal amounts of BANCR-overexpressing ACHN cells and the control cells were subcutaneously injected into nude mice. The tumor volumes were monitored and measured twice per week following tumor formation. When the mice were euthanized, the tumors were excised and their weights were determined. The results indicated that the tumor volume and weight formed by the BANCR-overexpressing ACHN cells were notably smaller than those formed by the control cells (Fig, 8, *A*–*C*). Similarly, equal amounts of the BANCR-knockdown 786-O cells and their negative control cells were subcutaneously injected into nude mice. The results showed that the tumor volume and weight formed by the BANCR-knockdown 786-O cells were markedly larger than those formed by the negative control cells (Fig. 8, *D–F*). We further analyzed the expression levels of BANCR in tumor tissues extracted from mouse, and the results are depicted in Figure 8*G*. The BANCR levels in each group of tumors were negatively correlated with tumor size. Thus, our animal studies strongly confirm the inhibitory effect of BANCR on ccRCC cell growth *in vivo*.

In addition, we evaluated the expression level and activity of G6PD in mouse tumor tissues as well as the content of R-5P. The results showed no significant change in G6PD expression in tumors derived from BANCR-overexpressing ACHN cells or BANCR-knockdown 786-O cells (Fig. 8, *H–J*). However, as illustrated in Figure 8, *K* and *L*, in the *in vivo* environment, when BANCR was overexpressed, both the activity of G6PD and the content of R-5P were reduced; conversely, when BANCR was knocked down, both greatly increased. Taken together, the results of *in vivo* experiments further supported that BANCR OE inhibited G6PD activity through post-transcriptional regulatory mechanisms, thereby limiting the tumor growth of ccRCC cells.

### G6PD was involved in the BANCR-mediated tumor-suppressive role

The accumulated evidence strongly suggests that BANCR may exert its tumor-suppressive effect on ccRCC through interaction with G6PD. To further explore this relationship, we conducted a series of experiments aimed at determining whether BANCR-mediated ccRCC cell proliferation and apoptosis depend on its interaction with G6PD. For this purpose, we used ACHN cells overexpressing BANCR (ACHN-BCR OE) and their corresponding control cells for rescue and reversal analysis.

**Figure 9:**
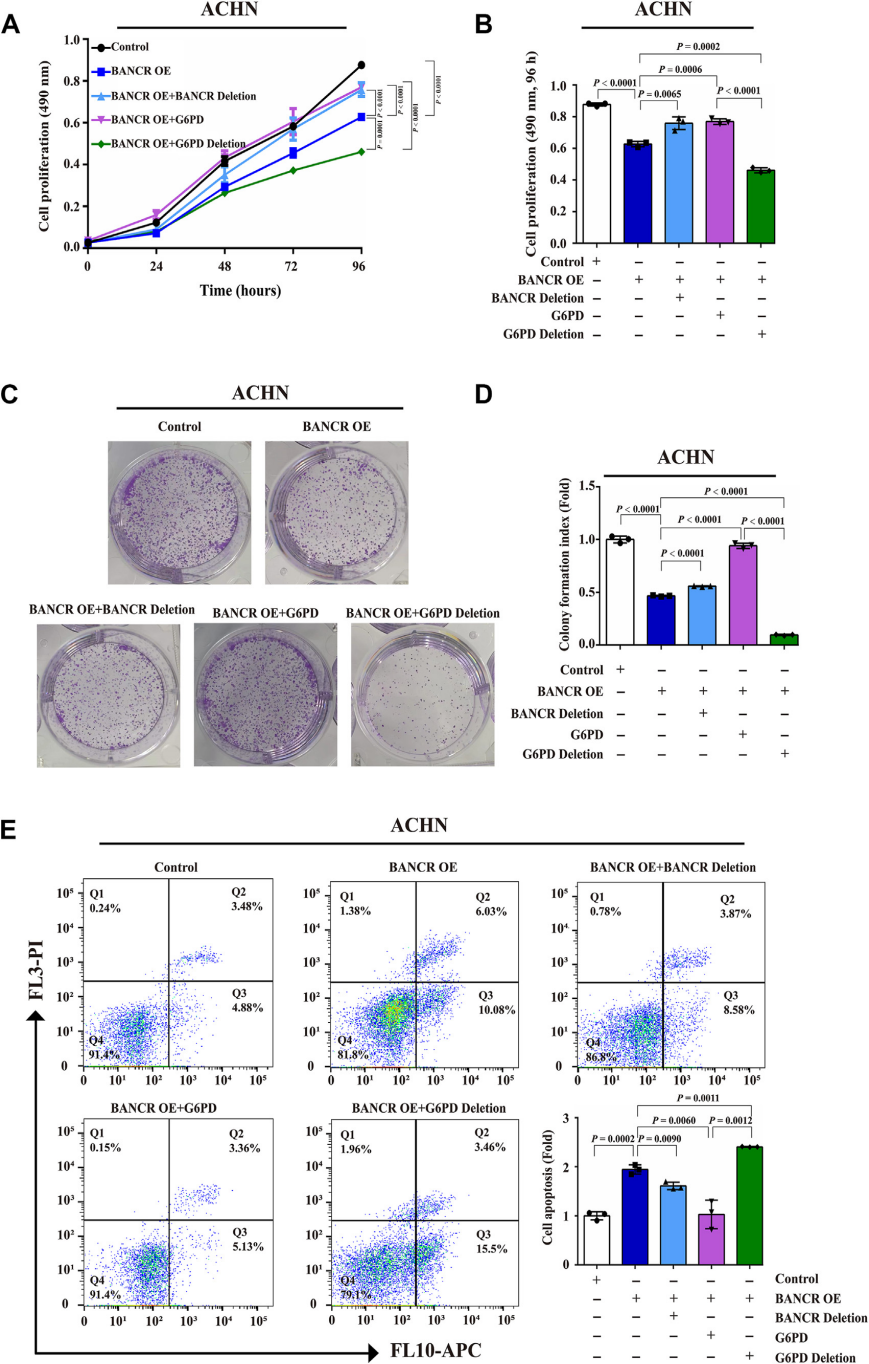
**BANCR mediated ccRCC proliferation and apoptosis through interacting with G6PD.***A*–*D*, the proliferation rates of ACHN-BANCR OE, ACHN-BANCR OE+BANCR Deletion, ACHN-BANCR OE+G6PD, ACHN-BANCR OE+G6PD Deletion, and relevant control cells were determined by MTS analyses (*A* and *B*), colony-formation assays, and quantitative evaluations (*C* and *D*). *E*, the apoptosis rates of ACHN-BANCR OE, ACHN-BANCR OE+BANCR Deletion, ACHN-BANCR OE+G6PD, ACHN-BANCR OE+G6PD deletion, and relevant control cells were measured by flow cytometry. All analyses were conducted at least three times. The data were shown as mean ± SD of three independent experiments. *A*, mixed ANOVAs were used, whereas unpaired Student's *t* tests were used for other analyses. BANCR, BRAF-activated noncoding RNA; ccRCC, clear cell renal cell carcinoma; G6PD, glucose 6-phosphate dehydrogenase; MTS, 3-(4,5-dimethylthiazol-2-yl)-5-(3-carboxymethoxyphenyl)-2-(4-sulfophenyl)-2H-tetrazolium; OE, overexpression.

First, the cell proliferation rate was assessed through MTS (3-(4,5-dimethylthiazol-2-yl)-5-(3-carboxymethoxyphenyl)-2-(4-sulfophenyl)-2H-tetrazolium) assay and colony-formation analysis. The results in Figure 9*A* showed that OE of BANCR significantly reduced the proliferation of ACHN cells. Compared with the control group, the proliferation rate decreased by about 30% 96 h after inoculation (Fig. 9*B*). However, when transfected with BANCR lacking G6PD-binding sites or G6PD-overexpressing plasmid, the proliferation inhibition of ACHN-BANCR OE cells was notably rescued. Intriguingly, G6PD transfection lacking an effective G6PD-BANCR binding site 3 (G6PD deletion) did not restore cell proliferation but further reduced the proliferation rate (Fig. 9, *A* and *B*). Similarly, OE of BANCR led to a significant reduction in ACHN cell colony formation, whereas BANCR deletion and G6PD OE further reversed this phenomenon, but G6PD deletion exacerbated the reduction in colony formation. In addition, G6PD deletion completely eliminated the ability of G6PD OE to rescue BANCR-mediated suppression of ccRCC cell proliferation (Fig. 9, *A*–*D*). These results underscored the critical role of G6PD binding site 3 (426–477 amino acid region) in BANCR-mediated ccRCC cell proliferation.

In the analysis of apoptosis in ccRCC cells, we observed that OE of BANCR dramatically increased the apoptosis rate in ACHN cells. In BANCR-overexpressing ACHN cells, the increase in apoptosis rate was reversed by approximately 17% and 36%, respectively, after transfection with BANCR deletion and G6PD OE. Conversely, G6PD deletion not only failed to reverse BANCR OE–induced apoptosis but also significantly increased the apoptosis rate compared with other groups (Fig. 9, *E* and *F*). These findings indicated that G6PD and its effective binding sites to BANCR were crucial for enhancing BANCR-mediated apoptosis in ccRCC cells.

In conclusion, the evidence supported that modification to BANCR *via* deletion of G6PD-BANCR binding sites (BANCR deletion), G6PD OE, or deletion of G6PD-BANCR binding site 3 (G6PD deletion) regulated the effects of BANCR on ccRCC cell proliferation and apoptosis. This highlighted the critical role of the lncRNA–protein interactions between BANCR and G6PD. Overall, these results confirmed that G6PD was a pivotal downstream target of BANCR, playing a significant role in the tumor-suppressive function mediated by BANCR in ccRCC.

## Discussion

ccRCC is the most prevalent and aggressive subtype of RCC. In recent years, the incidence and mortality rates of RCC have been rising annually (1, 2). Consequently, developing effective treatment methods for ccRCC continues to be a significant challenge, and early detection through a comprehensive understanding of its pathogenic mechanisms is crucial for advancing RCC research. Recently, the role of lncRNA in RCC has garnered considerable attention (32). As a noncoding gene, lncRNA has been shown to be a pivotal regulatory factor in tumorigenesis and progression. BANCR, a 693 bp RNA located on chromosome 9, was initially identified in melanoma in 2012 through RNA sequencing screening for transcripts influenced by the oncogene BRAF^V600E^. Although BANCR is an important lncRNA associated with cancer, its expression patterns vary among various types of cancer, and its function exhibits a dual nature, acting both as a tumor suppressor and a tumor promoter (23). For instance, in bladder cancer and hepatitis B virus–related hepatocellular carcinoma, BANCR levels were notably diminished, making it a promising biomarker and therapeutic target for certain cancers (33). In contrast, in gastric cancer, elevated BANCR levels facilitated cancer cell proliferation by regulating NF-κB1 (34).

A previous study indicated a trend of BANCR downregulation in ccRCC; however, its specific role and potential regulatory mechanisms in this context remained unclear (27). In this study, we investigated the expression pattern of BANCR in ccRCC, elucidating its specific role and regulatory mechanism in the development of ccRCC. Our results demonstrated that BANCR expression in ccRCC was dramatically lower than in normal levels. Conversely, OE of BANCR effectively inhibited the proliferation of ccRCC cells and greatly promoted their apoptosis by regulating cell cycle and apoptosis-related molecules. Furthermore, OE of BANCR markedly inhibited the development of ccRCC *in vivo*. Although G6PD is localized on the X chromosome, no significant correlation was found between gender and the conclusion that BANCR OE limited tumor growth of ccRCC cells *in vivo*. These results strongly suggested that BANCR played a potential role in cancer suppression during the tumorigenesis of ccRCC. Further investigation revealed that BANCR could bind to G6PD through direct lncRNA–protein interaction and inhibit G6PD activity by restraining its dimer formation.

In addition, our study also discovered that downregulation of BANCR resulted in a shift in the glucose metabolism mode of ccRCC cells toward aerobic glycolysis and the PPP, likely achieved through interaction with the downstream target G6PD. Although this study marked the first revelation of the molecular mechanisms underlying the biological functions of BANCR in ccRCC, several critical questions remained unanswered. ccRCC is widely regarded as a metabolic disorder characterized by high pentose phosphate metabolism rates, the "Warburg effect," and rapid production of nucleotide, lipid, and amino acid synthesis precursors (8, 9, 10, 11, 12). Given that G6PD is one of the primary regulatory factors of ccRCC metabolic disorders and can be manipulated by BANCR, it is currently unclear to what extent BANCR is involved in the metabolic reprogramming of ccRCC or other cancers. This issue is crucial for further studies. In addition to determining the relationship between BANCR and metabolic reprogramming, further exploration is needed to investigate the reasons for the downregulation of BANCR during the progression of ccRCC as well as its potential other functions in ccRCC invasion, migration, and resistance to targeted therapy or immunotherapy.

G6PD is a pivotal mediator of metabolic reprogramming, influencing various cellular functions, such as proliferation, cell cycle regulation, apoptosis, and maintaining redox homeostasis (13, 14). Consequently, abnormal G6PD expression is widely believed to promote tumorigenesis of various human cancers (13, 14). Despite numerous studies demonstrating the crucial role of G6PD in tumorigenesis, the specific molecular mechanisms governing its expression and activity regulation in tumor cells remain elusive. Our research team's prior studies demonstrated that G6PD OE was not only a potential predictor of poor prognosis in ccRCC patients but was also positively associated with ccRCC development. Importantly, the high expression and overactivation of G6PD were attributed to transcriptional and post-translational regulatory mechanisms (6, 16, 18, 19). As a key enzyme in metabolism, the carcinogenic effect of G6PD was closely tied to its enzymatic activity, which was only manifested in its dimer or tetramer conformation. Reports suggested that G6PD OE in hepatocellular carcinoma or ccRCC was mediated by Nrf2 or through synergistic overactivation of the NF-κB and pSTAT3 signaling pathways, respectively (18, 35). Meanwhile, G6PD activity's dimerization could be promoted through protein–protein interaction with Polo-like kinase 1 (PIk1) at the post-transcriptional level, whereas formation of complexes with p53 greatly inhibited this process (36, 37). PIk1, acting as a cell mitotic regulator, directly binds to G6PD, facilitating the formation of its active dimer and activation of the PPP, thereby driving cell cycle progression and tumor cell growth (36). In contrast, research revealed that p53 could directly bind to G6PD, preventing its dimer formation and thus inhibiting its enzymatic activity and the PPP (37).

The aforementioned evidence suggested that kinases or other cofactors could manipulate the activity of G6PD at the post-translational level by regulating the multimeric conformation of G6PD. However, there are currently no reports on whether G6PD can directly bind to or be regulated by some lncRNAs. Most importantly, in the study of ccRCC, we have confirmed that the highly expressed SIRT2 can interact with G6PD, facilitate its activity through deacetylation, and increase its stability by reducing ubiquitination and enhancing SUMO1 modification, leading to an increase in G6PD activity and tumorigenesis in ccRCC (19). However, the specific reasons for G6PD overactivation in ccRCC are not yet clear. In this study, we demonstrated that BANCR could bind to G6PD through direct lncRNA–protein interaction and downregulate its activity by restraining the formation of G6PD dimers. This discovery revealed G6PD dysregulation and its potential mechanisms in clinical ccRCC tumorigenesis. In addition, numerous studies have confirmed that changes in G6PD dimer formation can affect the integrity of this active enzyme (21, 36, 38), which is consistent with our previous and present results (19). We found that OE of SIRT2 or knockdown of BANCR did not obviously alter the expression of G6PD, but they could inhibit its activity by suppressing the formation of G6PD dimers. This may indicate that other transcriptional, translational, or post-translational regulatory mechanisms may have synergistic or compensatory effects on maintaining high expression of G6PD, which needs further exploration in ccRCC.

Overall, this study expanded our understanding of the key role of BANCR. The findings suggested that functional BANCR might become a promising biomarker for cancer diagnosis and prognostic evaluation. Interventions targeting BANCR may also become a novel therapeutic strategy for ccRCC. Furthermore, we provided a detailed description of the precise interaction between BANCR and G6PD. Our R-IP assay results showed that the 276 to 327 nt region of BANCR could bind to the 426 to 477 amino acid region of G6PD, regulating the formation and activity of G6PD dimers *via* direct lncRNA–protein interactions. It has been confirmed that the amino acid regions 51 to 102 and 90 to 141 of the G6PD protein are located within its "catalytic" NADP^+^ coenzyme–binding domain, which is crucial for enzyme activity. Meanwhile, the 426 to 477 region is close to the "structural" NADP^+^ site and located near the G6PD dimerization interface (29). Changing certain amino acid residues on the interface, or the interaction between G6PD and specific regulatory factors, can affect the dimerization and enzyme activity of G6PD (21, 30). Our current research findings have identified the most likely binding domains for BANCR and G6PD. Although this model has been outlined, the reason for the change of G6PD activity in other human cancers remains elusive.

Research indicates that ccRCC is a metabolically heterogeneous disease (9, 10, 11, 12, 28). It has been reported that ccRCC can be categorized into three subtypes: GP1, characterized by immune infiltration; GP2, marked by metabolic remodeling; and GP3, distinguished by stromal components (28). Given that the ratio of decreased to increased expression of BANCR in ccRCC is approximately 2:1, further research is essential to comprehensively elucidate whether BANCR assumes similar or distinct roles across different subtypes of ccRCC. In this study, three shRNA-loaded lentiviral vectors were employed to knock down BANCR expression. However, different shRNAs, especially BANCR KD2 and BANCR KD3, showed consistent trends in the knockdown efficiency of BANCR, cell phenotypes, and phenotype-related molecular markers in different cell lines as compared with the control, but there were certain differences in specific values. To further validate our findings, we will design additional shRNAs and employ CRISPR–Cas9-mediated genome deletion to knockout lncRNA BANCR, thereby corroborating the shRNA results in our further research. Moreover, given the pivotal role of G6PD as a metabolic regulator, it is reasonable to postulate the involvement of BANCR-mediated G6PD overactivation in the comprehensive metabolic reprogramming of ccRCC. In summary, this study delved into the roles and mechanisms behind BANCR and G6PD dysregulation, providing new research directions for further exploration of the pathogenesis and progression of ccRCC.

## Conclusion

**Figure 10:**
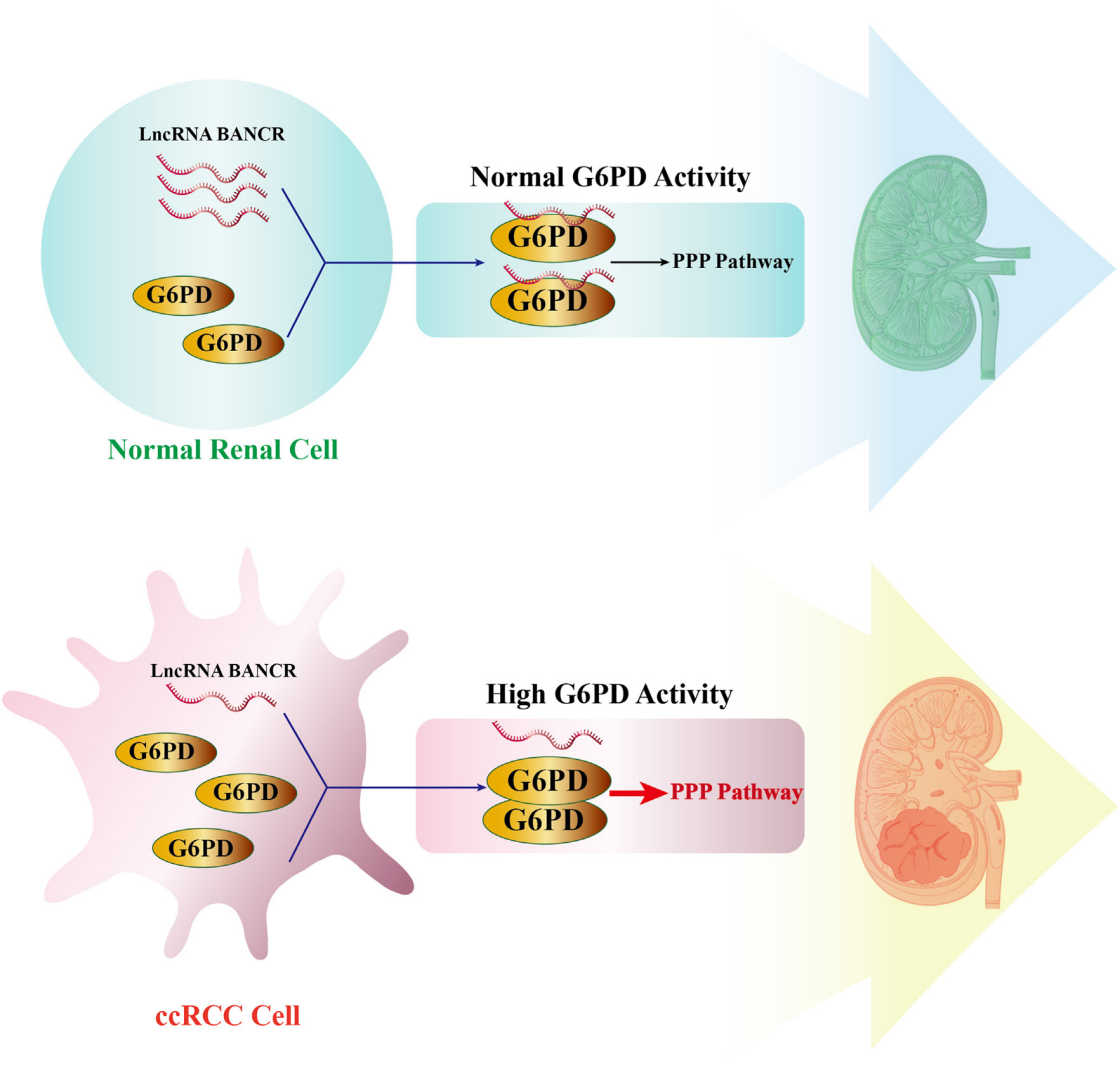
**LncRNA BANCR attenuated ccRCC tumorigenesis *via* repression of G6PD activity.** LncRNA BANCR was downregulated in ccRCC and significantly impacted its tumorigenesis. The underlying mechanism involved BANCR binding to G6PD through direct lncRNA–protein interaction, thereby inhibiting G6PD activity by restraining dimer formation. BANCR, BRAF-activated noncoding RNA; ccRCC, clear cell renal cell carcinoma; G6PD, glucose 6-phosphate dehydrogenase; lncRNA, long noncoding RNA.

Collectively, the objective of this study was to elucidate the role of BANCR (lncRNA BANCR) in ccRCC tumorigenesis and to reveal the potential mechanism by which LncRNA BANCR inhibited G6PD activity, thereby mitigating ccRCC tumorigenesis. The research results indicated that lncRNA BANCR exhibited a downregulation trend in ccRCC, which modulated cell survival by affecting cell proliferation and apoptosis. The potential mechanism involved BANCR binding to G6PD through direct lncRNA–protein interactions, thereby reducing G6PD activity by restraining dimer formation. Moreover, BANCR also regulated glucose metabolism flow within ccRCC cells. In addition, OE of BANCR inhibited the growth of ccRCC cells *in vivo*, and G6PD was implicated in BANCR-mediated tumor suppression (Fig. 10). In summary, this study offered fresh insights into the molecular pathogenesis of ccRCC, unveiling a unique and novel regulatory mechanism by which G6PD's ectopic overactivation contributed to the progression of ccRCC. It was believed that BANCR-mediated inhibition of G6PD activity could potentially become a promising treatment strategy for ccRCC.

## Experimental procedures

### Clinical tissue specimens

Samples of fresh ccRCC specimens and adjacent normal renal tissues were obtained from the patients with primary ccRCC who underwent surgery between 2014 and 2017 at the Department of Organ Transplantation of the First Affiliated Hospital of Kunming Medical University. All these tissues were immediately frozen and stored in liquid nitrogen. These experiments were performed in accordance with the regulations of Helsinki Declaration and approved by the Ethics Committee of Kunming Medical University with patients providing signed informed consent.

### Stable cell line establishment

The most used cell lines for ccRCC researches, including ACHN, 786-O, Caki-1, were obtained from Chinese Academy of Sciences, Kunming Institute of Zoology, 293T cells were provided by Guangzhou Leader Biotechnology Co, LTD, and all cells were validated by short tandem repeat profiling. All cells were mycoplasma free and cultured in Dulbecco's modified Eagle's medium (catalog no.: 0024518, BI) containing 10% fetal bovine serum (catalog no.: 1818398, BI) in 37 °C humidified incubator (5% CO_2_).

To construct stable BANCR–overexpressing and BANCR-knockdown cell lines, ACHN and 786-O cells were seeded in a 6-well plate. When the cells reached approximately 70% confluence, they were transfected with either LV5-NC (OE Control) or LV5-homo lncRNA BANCR (BANCR OE) to establish stable BANCR–overexpressing cell lines. To establish stable BANCR knockdown cell lines, cells were transfected with either LV3-NC (KD Control, sequence, 5′-TTCTCCGAACGTGTCACGT-3′) or LV3-homo LncRNA BANCR knockdown (BANCR KD1 sequence, 5′-CTTGGTCAGAGGTTGGATGAA-3′; BANCR KD2 sequence, 5′-GGAGTGGCGACTATAGCAAAC-3′; BANCR KD3 sequence, 5′-TTCTAATTCTGAGCCTCTATT-3′). All lentivirus were customized by GenePharma. After 48 h infection, the cells were treated with puromycin (5 μg/ml) selection for 15 days, and the puromycin-containing culture medium was changed every 2 days. GFP expression levels were determined under fluorescence microscope, and real-time RT–PCR was performed to identify these stable transfected cells.

### Real-time RT–PCR

Total RNA extraction was performed by using Trizol reagent (catalog no.: 9109; TakaRa) and phenol–chloroform–isopentyl alcohol (catalog no.: P1011; Solarbio), and synthesis of complementary DNA was conducted by using RevertAid First Strand cDNA Synthesis Kit (catalog no.: K1622; Thermo) according to the manufacturers' instruction. Real-time RT–PCR was performed by using FastStart Universal SYBR Green Master (catalog no.: 04913914001; Roche). Primer sequences are shown in Table 1.

**Table 1:** Sequences of primers used for real-time RT–PCR analysis

Primers	Primer sequences (5′–3')
U6	Forward: CTCGCTTCGGCAGCACA
	Reverse: AACGCTTCACGAATTTGCGT
BANCR	Forward: ATCTCACCTCTGCAAAGAGCA
	Reverse: CAATGTGGTGCCAGGGATGA
G6PD	Forward: TCATCATCATGGGTGCATCGG
	Reverse: CTTGAAGAAGGGCTCACTCTGTTTG
Cyclin D1	Forward: GCTGCGAAGTGGAAACCATC
	Reverse: CCTCCTTCTGCACACATTTGAA
CDK4	Forward: TCAGCCAGCTTGACTGTTCCA
	Reverse: GCCTAGATTTCCTTCATGCCA
CDK6	Forward: TGACCAGCAGCGGACAAATAA
	Reverse: TGTACCACAGCGTGACGACCA
Cyclin E1	Forward: ACTCAACGTGCAAGCCTCG
	Reverse: GCTCAAGAAAGTGCTGATCCC
CDK2	Forward: CCAGGAGTTACTTCTATGCCTGA
	Reverse: TTCATCCAGGGGAGGTACAAC
Bcl2	Forward: GTGCCTGCTTTTAGGAGACCGA
	Reverse: GAGACCACACTGCCCTGTTGATC
BAX	Forward: AGACACTCGCTCAGCTTCTTG
	Reverse: CTTTTGCTTCAGGGTTTCATC

### Western blot analysis

Cell lysates were prepared, and Western blot analysis was performed as previously described (17). Following reagents and antibodies were used: RIPA lysis buffer (catalog no.: R0010; Beijing Solarbio Science & Technology Co, Ltd), polyvinylidene difluoride membrane (catalog no.: IPVH00010; Millipore), anti-G6PD antibody (catalog no.: ab993; Abcam), anti-β-actin antibody (catalog no.: 0061R; Bioss), anti-Bcl-2 antibody (catalog no.: ab32124; Abcam), anti-Bax antibody (catalog no.: Ab182733; Abcam), anti-MMP2 antibody (catalog no.: Ab180116; Abcam), anti-MMP9 antibody (catalog no.: Ab228402; Abcam), anti-MMP13 antibody (catalog no.: Ab51072; Abcam), anti-CyclinD1 antibody (catalog no.: Ab134175; Abcam), anti-CDK4 antibody (catalog no.: Ab68266; Abcam), anti-CDK6 antibody (catalog no.: Ab288368; Abcam), anti-CyclinE1 antibody (catalog no.: Ab224819; Abcam), anti-CDK2 antibody (catalog no.: Ab235941; Abcam), anti-P27 antibody (catalog no.: ab32034; Abcam), anti-PCNA antibody (catalog no.: Ab18197; Abcam), and anti-rabbit goat IgG antibody (catalog no.: ab6721; Abcam).

### Cell apoptosis detection

Cell apoptosis was determined by using the Apoptosis Assay Kit (catalog no.: AD11; DOJINDO) and flow cytometry analysis. Cells were first seeded in a 6-well plate and treated for apoptosis induction. When they reached about 70% confluence, the cells were digested with trypsin free of EDTA. After washing with PBS for three times, the cells were resuspended with 100 μl 1× Annexin V Binding Solution, adding 5 μl Annexin V 633 and 5 μl propidium iodide, respectively, and then mixed. Ten thousand cells were detected by flow cytometry, and the results were analyzed by Flojo7.6 software. For TUNEL assay, one-step TUNEL apoptosis assay Kit (catalog no.: C1090; Beyotime) was used according to the manufacturers' protocol.

### Cell proliferation and colony-formation assay

MTS cell proliferation assay kit (catalog no.: CTB169; Promega) was used for the cell proliferation assay following the manufacturers' protocol and as our previous description (19). For colony-formation analysis, 2 × 10^3^ ccRCC cells were seeded in a 6-well plate, cultured for 10 days, and stained with crystal violet for colony counting. Cell colony with a cell count greater than 50 is counted, and quantification assessed under a microscope. The cell cycle assays were conducted by inoculating cells into a 6-well plate, followed by digestion and centrifugation. Subsequently, the cells were slowly dropwise added to precooled 75% ethanol and fixed at 4 °C for 24 h. Then the cells were collected through centrifugation, stained with propidium iodide/RNase staining kit (catalog no.: 550825; BD Pharmingen), and subjected to flow cytometry detection using BD FACSCelesta flow cytometry with results being analyzed using Flojo7.6 software.

### G6PD activity detection

G6PD activities of ACHN-BANCR OE, 786-O-BANCR KD, and relevant control cells or xenografted nude mice tissues of each group were detected using the G6PD assay kit (catalog no.: GMS70013.1; GenMed). All these analyses were carried out according to the manufacturers' instructions and as described previously (17).

### Glutaraldehyde cross-linking assay

After harvest, the cells were resuspended using PBS with glutaraldehyde added to a concentration of 0.025% and incubated at room temperature for 1 h. The formation of G6PD monomer and dimer was determined by Western blot assay and grayscale scanning analyses.

### Ribose-5-phosphate content assay

The R-5P levels were determined using the R-5P ELISA Kit (catalog no.: MM-63490H1; Meimian). Cells were harvested and lysed, and their quantity was measured by the bicinchoninic acid method. Subsequently, all samples were standardized to equal masses and volumes. Following the manufacturer's instructions, 40 μl of sample diluent and 10 μl of the sample were added to each well, thoroughly mixed, and incubated at 37 °C for 30 min. The plate was washed five times with washing solution before adding 50 μl of horseradish peroxidase conjugate reagent, which was then incubated at 37 °C for another 30 min. After washing again, chromogen solution was added and incubated at 37 °C for a duration of 10 min. Finally, stop solution (50 μl) was added, and the absorbance at a wavelength of 450 nm was measured using a microtiter plate reader.

### RNA FISH assay

RNA FISH was performed using the RNA FISH kit with SA-Biotin system (Genepharma) according to the manufacturers' instructions. For BANCR probe hybridization in ccRCC cells: a total of 1 × 10^4^ ccRCC cells were cultured in a 48-well plate with cover slide and incubated overnight. Each well was fixed with 4% paraformaldehyde for 15 min at room temperature, followed by treatment with 0.1% buffer A for another 15 min. After blocking the cells at 37 °C for 30 min, they were treated with 2× buffer C and incubated again at the same temperature for another 30 min. The probe was diluted according to the manufacturer's instructions, denatured in a water bath at 75 °C for 10 min, and then added proportionally to PBS with SA-Cy3 before being incubated at 37 °C for 30 min. The probe working solution was mixed with buffer E and added to cell culture wells where it hybridized overnight in a cell culture incubator. The probe mixture was aspirated and discarded on the following day, followed by addition of 0.1% buffer F to each well for washing at 37 °C for 10 min. Subsequently, the wells were washed with 2× buffer C at both 60 °C and 37 °C for a duration of 10 min each, followed by three additional washes.

For BANCR probe hybridization in paraffin sections: the paraffin sections were baked at 60 °C for 2 h. Subsequently, the sections were immersed in xylene I and II for a duration of 10 min each, followed by incubation in graded alcohol (100%, 95%, 90%, 80%, and 70%) for a period of 10 min each at room temperature. The proteinase K working solution was prewarmed to cover the sections and then incubated at a temperature of 37 °C for a duration of 20 min. A blocking solution was added dropwise to each section and incubated at a temperature of 37 °C for a period of 30 min. Finally, the sections were covered with drops of buffer C (2×) and rinsed three times at room temperature for 1 min each. The preheated denaturing solution was added dropwise onto the sections and incubated at 78 °C for 8 min. The gradient alcohol was dehydrated sequentially at 70%, 80%, 90%, and finally, 100% for a duration of 2 min each time, followed by air drying. The probe working solution and buffer E were thoroughly mixed and applied to cover the sections, which were then placed in an *in situ* hybridizer and incubated at a temperature of 37 °C for 12 h. Following hybridization, the sections were subjected to dropwise washing with preheated washing solution for a duration of 15 min. Subsequently, they underwent three rounds of washing with buffer C at temperatures of both 60 °C and then subsequently at 37 °C, each round lasting for a duration of 10 min.

For G6PD staining and immunofluorescence analyses, ccRCC cells and the paraffin sections were incubated with 100 μl of G6PD antibody (catalog no.: sc-373886; Santa Cruz Biotechnology, Inc) overnight at 4 °C. After PBS washing, a secondary antibody labeled with fluorescein was added and incubated at room temperature for 2 h. Finally, the antiquenching agent (containing 4′,6-diamidino-2-phenylindole) was added and examined using a fluorescence microscope.

### R-IP assay

R-IP detection was conducted using the Magna RIP TM RNA-Binding Protein Immunoprecipitation Kit (catalog no.: 17-700; Millipore) according to the manufacturers' instructions. Briefly, 5 μg anti-G6PD antibody (catalog no.: ab993; Abcam) or goat anti-rabbit IgG antibody (catalog no.: ab6721; Abcam) was initially added to 50 μl of pretreated magnetic beads and incubated in a rotating culture for 30 min at room temperature. Then, 1 × 10^7^ ccRCC cells were treated with 200 μl RIP lysate and centrifuged at 14,000 rpm for 10 min at 4 °C. Next, 100 μl of supernatant was added to the beads–antibody complex containing 900 μl of RIP buffer and placed on a rotary to incubate at 4 °C overnight. Subsequently, proteinase K was introduced and incubated for 30 min at 55 °C with shaking to digest the proteins, followed by addition of phenol:chloroform:isoamyl alcohol for RNA purification. Finally, real-time RT–PCR analysis was performed to detect the recruitment level of BANCR to G6PD protein.

### ECAR and glycoPER assays

The ECAR and glycoPER using Seahorse XF Glycolysis Rate Assay Kit (catalog no.: 103344-100; Agilent Technologies) and the Seahorse XFe 24 analyzer. Briefly, a total of 1.5 × 10^4^ BANCR overexpressing, BANCR knocked down, as well as relevant control cells were respectively harvested. Subsequently, these cells were incubated in a solution containing 10 mM glucose, 2 mM glutamine, 1 mM sodium pyruvate, and Hepes buffer in the Seahorse XF Glycolysis Rate Assay solution and incubated in a CO_2_-free incubator for 40 min. After obtaining baseline measurements, cell plates were transferred to the Seahorse XFe24 analyzer where each well was automatically filled with 0.5 μM of Rot/AA and 10 mM of 2-deoxy-d-glucose. The data obtained were then analyzed using Seahorse XF24 Wave software.

### Glucose metabolite analysis

ACHN-BANCR OE and relevant control cells (1 × 10^7^) were subjected to targeted glucose metabolomics analysis by Shanghai Applied Protein Technology. Briefly, samples were prepared for UHPLC-QTRAP MS analysis on AB 6500+ Q-trap (AB SCIEX) and Agilent 1290 Infinity LC system (Agilent) according to the protocol. MultiQuant was used for quantitative data processing.

### Glycolysis analysis

For ccRCC cells, glycolysis analysis, glucose uptake, and lactate production of stably transfected ccRCC cells was detected. About 1 × 10^5^ cells were first cultured in a 6-well plate and harvested at 48 h after seeding. Glucose Uptake Colorimetric Assay Kit (catalog no.: K676-100; Biovision) and a Lactate Colorimetric Assay Kit (catalog no.: K627-100; Biovision) were used for glucose and lactate concentration detection according to the manufacturer’s protocols.

For the glucose uptake assay, Reaction Mix A (10 μl/well) was prepared and added to each reaction according to the manufacturer's instructions. The reactions were then incubated at 37 °C for 1 h. Subsequently, Extraction Buffer I (90 μl) was added to each well. The microtiter plate was sealed with an aluminum seal and incubated at 90 °C for 40 min, followed by cooling on ice for 5 min. Neutralization Buffer II (12 μl) was added to neutralize the reaction in each well, and Reaction Mix B (38 μl) was prepared for each reaction. Finally, absorbances were measured at 412 nm using a U-1800 ultraviolet spectrophotometer (Agilent, BioTek, Synergy H1), every 2 to 3 min at a temperature of 37 °C.

For lactate concentration assays, a reaction mixture of 50 μl was prepared for each reaction. The mixture was added to each well, thoroughly mixed, and incubated at room temperature for 30 min in accordance with the manufacturer's instructions. Subsequently, absorbances were measured at OD 450 nm using a U-1800 ultraviolet spectrophotometer (Agilent, BioTek, Synergy H1).

### Xenografted animal model

Equal numbers of 6-week-old female and male BALB/c nude mice (n = 6 per group) were injected s.c. with 1 × 10^7^ ACHN-BANCR OE, 786-O-BANCR KD2, or relevant control cells suspended in 200 μl PBS into the mice oxter flank. Tumor volumes and weight were calculated as described previously (17). Mice were euthanized by pentobarbital sodium injection, and tumor samples were harvested for subsequent analysis. All animal experiments were approved by the Institutional Animal Care and Use Committee of Kunming Medical University.

### Statistical analysis

Data of the BANCR expression profile in ccRCC and control tissues were downloaded from TCGA and analyzed using the Mann–Whitney *U* test. The experimental data were analyzed by GraphPad Prism 6.0 software (GraphPad Software, Inc) and expressed as means ± SD. The differences between groups were compared using the Student's *t* test (two groups) or one-way ANOVA (multiple groups). Comparisons in MTS assay and xenografted mouse models were determined using mixed ANOVA. A *p* value of less than 0.05 meant that difference was statistically significant. Graphs were designed using GraphPad Prism 6.0.

## Data availability

The data used to support the findings of this study are available from the corresponding author upon request.

## Ethics approval and consent to participate

All experiments involving human participants were performed in accordance with the regulations of the Helsinki declaration and approved by the Ethics Committee, Kunming Medical University. All patients provided prior written informed consent. All animal experiments were performed according to the Regulations for the Administration of Affairs Concerning Experimental Animals (China, 1988) and approved by the Institutional Animal Care and Use Committee, Kunming Medical University.

## Supporting information

This article contains supporting information.

## Conflict of interest

The authors declare that they have no conflicts of interest with the contents of this article.
