# Supplementary material for: A BRAF-activated noncoding RNA attenuates clear cell renal cell carcinoma via repression of glucose-6-phosphate dehydrogenase
Source: J Biol Chem. 2025 Jan 31;301(3):108247. doi: 10.1016/j.jbc.2025.108247 (PMC11889594; doi:10.1016/j.jbc.2025.108247)
Supplement: Supplementary data 1 [file mmc1.zip › Supplementary data 1-STR identification certificates for cell lines/293T.pdf]

Cell Line Authentication Service

STR Profiling Report

Sample Code: 293T

Sample Type: Cell Line

Testing Method: STR Genotyping

Report Time: October 20, 2023

Add: Room 508, NO.1015 Longteng Rd , Songjiang District, Shanghai/ Tel:+86-18521538068 / E-mail: zhusn@biowing.com.cn

COMPANY STATEMENT

1. THIS REPORT IS ONLY RESPONSIBLE FOR THE SAMPLES ANALYZED.
2. THE TESTING RESULTS AND THE ORGANIZATION NAME WILL NOT BE USED FOR ADVERTISEMENT, COMMERCIAL EXHIBITIONS, COMMERCIAL PERFORMANCE AND OTHER COMMERCIAL ACTIVITIES.
3. OBJECTIONS SHOULD BE RAISED WITHIN FIFTEEN DAYS AFTER THE RECEIPT OF THIS REPORT.
4. THE PAPER REPORT WITH CONTENT ALTERING, ADDING OR WITHOUT THE STAMPED SEAL OF THE COMPANY ARE INVALID.

Testing Company: Shanghai Biowing Applied Biotechnology Co. Ltd

Address: Room 502, NO.1015 Longteng Rd , Songjiang District, Shanghai

Tel: +86-18521538068

Contact: Shuangning Zhu

E-mail: zhusn@biowing.com.cn

Cell Line Authentication – STR Profiling Report

Sample code

Table 1. Sample Code

Customer's code	Company Code
293T	20231017-01

Sample Number :1

Sample Type: Cell line

Testing Type: STR

Testing Method:

DNA was extracted by a commercial kit from CORNING (AP-EMN-BL-GDNA-250G). The twenty STRs including Amelogenin locus were amplified by six multiplex PCR and separated on ABI 3730XL Genetic Analyzer. The signals were then analyzed by the software GeneMapper.

Data Interpretation:

Cell lines were authenticated using Short Tandem Repeat (STR) analysis asdescribed in 2012 in ANSI Standard (ASN-0002) by the ATCC Standards Development Organization (SDO) and in Capes-Davis et al., Match criteria for human cell line authentication: Where do we draw the line? Int J Cancer.2013;132(11):2510-9.

Add: Room 508, NO.1015 Longteng Rd , Songjiang District, Shanghai/ Tel:+86-18521538068
/ E-mail: zhusn@biowing.com.cn

Test Results

1. STR profile

Table 2. STR and Amelogenin Genotyping Results of Cell line.

Loci	Sample information			Cell Bank information		
	Sample name：293T			Cell line name：HEK293T		
	**Allele1**	**Allele2**	**Allele3**	**Allele1**	**Allele2**	**Allele3**
D5S818	8	9		8	9	
D13S317	12	12		12	12	
D7S820	11	11		11	11	
D16S539	9	13		9	13	
VWA	16	19		16	19	
TH01	7	9.3		7	9.3	
AMEL	X	X		X	X	
TPOX	11	11		11	11	
CSF1PO	11	12		11	12	
D12S391	19	21				
FGA	23	23				
D2S1338	19	19				
D21S11	28	30.2				
D18S51	17	18				
D8S1179	11	12	14			
D3S1358	15	16	17			
D6S1043	11	11				
PENTAE	7	15				
D19S433	17	18.2				
PENTAD	9	10				
D1S1656	15	17.3				

Add: Room 508, NO.1015 Longteng Rd , Songjiang District, Shanghai/ Tel:+86-18521538068 / E-mail: zhusn@biowing.com.cn

2. database annotation

Figure 1. STR matching analysis

EV	Cell No.	Cell name	Locus names								
			D5S818	D13S317	D7S820	D16S539	VWA	TH01	AM	TPOX	CSF1PO
	Query (Your Cell)		8,9,	12,12,	11,11,	9,13,	16,19,	7,9.3,	X,X,	11,11,	11,12,
1.0(36/36)	CVCL_0063	HEK293T	['8', '9']	['12', '12']	['11', '11']	['9', '13']	['16', '19']	['7', '9.3']	['X', 'X']	['11', '11']	['11', '12']
0.94(34/36)	RCB2202	293T	['8', '9']	['12', '14']	['11', '11']	['9', '13']	['16', '19']	['7', '9.3']	['X', 'X']	['11', '11']	['11', '12']

Note: The STR online match analysis of the test cell against DSMZ/ATCC/EXPASY database, showing cell number (Cell No.) and cell name.

3. Authentication

☐ The submitted sample profile is human, but not a match for any profile in the DSMZ STR database.

☑ The submitted profile is exact match for the following human cell line(s) in the EXPASY STR database (8 core loci plus Amelogenin): **HEK293T**.

☐ The submitted profile is similar to the following DSMZ human cell line: /.

- **Note:** Cell lines are considered be related, derived from a common ancestry, when 80% (exact match) of the alleles in its STR profile match profiles from tissue or other cell line samples from that donor or from database. Cell lines with between a 55% to 80% (similar) match require further profiling for investigation of relatedness.

Figure 2. STR profiles of sample cell line

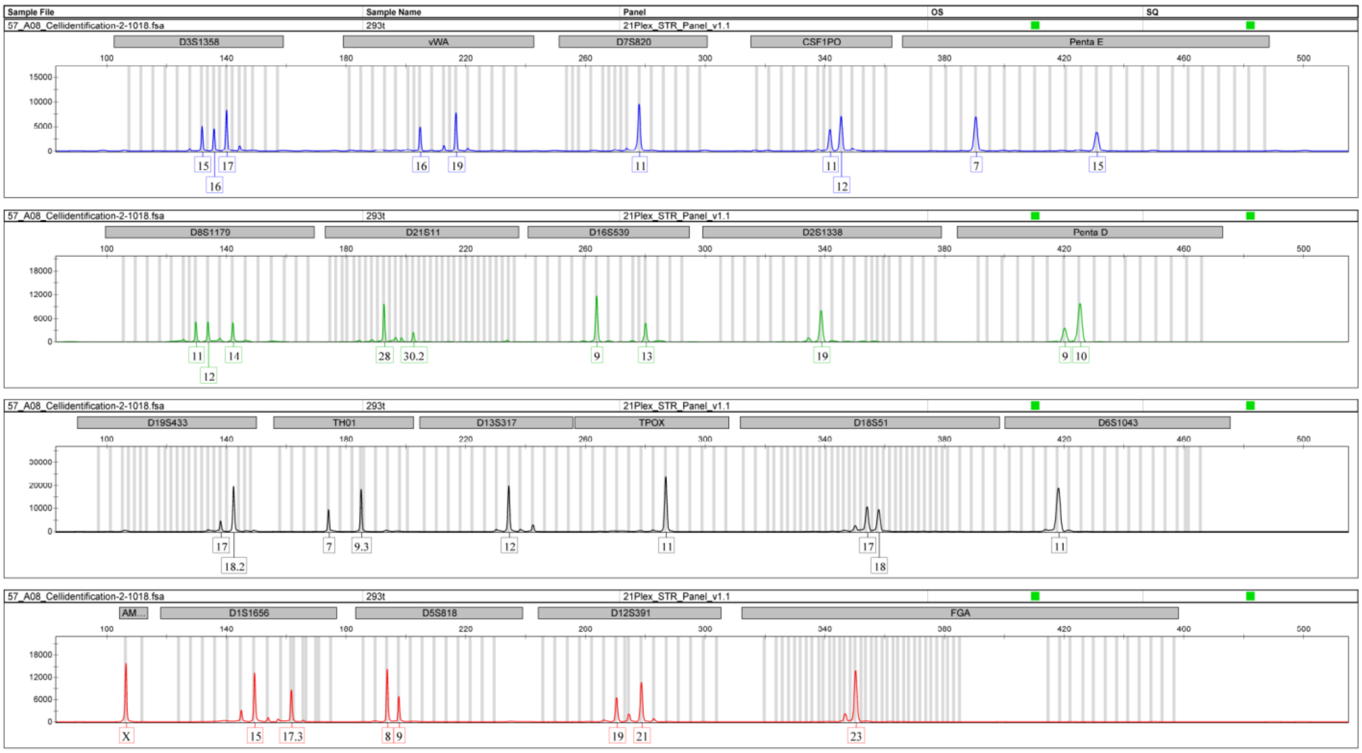

Add: Room 508, NO.1015 Longteng Rd , Songjiang District, Shanghai/ Tel:+86-18521538068 / E-mail: zhusn@biowing.com.cn

Appendix

1. Genotyping Strategy and Site Distribution

Table S1. Experimental Strategy and Sites

	Strategy 1	Strategy 2	Strategy 3	Strategy 4
1	D3S1358	D8S1179	D19S433	AMEL
2	VWA	D21S11	TH01	D1S1656
3	D7S820	D16S539	D13S317	D5S818
4	CSF1PO	D2S1338	TPOX	D12S391
5	PENTAE	PENTAD	D18S51	FGA
6			D6S1043	

The allele match algorithm compares the 8 core loci plus amelogenin only, even though alleles from all lociwill be reported when available.

2. DSMZ tools was used to carry on the cell line comparison, which contains 2455 cell lines STR data from ATCC, DSMZ, JCRB ,ECACC， GNE and RIKEN databases. If the cell is not included in the above cell library, users need to compared with other databases.

STR Profiling
Cell Line Authentication

Technician: Jianan Zhang

Checked by: Ning Qian

Issued by: Yang Bai

Issue date: October 20, 2023

Add: Room 508, NO.1015 Longteng Rd , Songjiang District, Shanghai/ Tel:+86-18521538068 / E-mail: zhusn@biowing.com.cn
