# Supplementary figures and images for: A BRAF-activated noncoding RNA attenuates clear cell renal cell carcinoma via repression of glucose-6-phosphate dehydrogenase
Source: J Biol Chem. 2025 Jan 31;301(3):108247. doi: 10.1016/j.jbc.2025.108247 (PMC11889594; doi:10.1016/j.jbc.2025.108247)

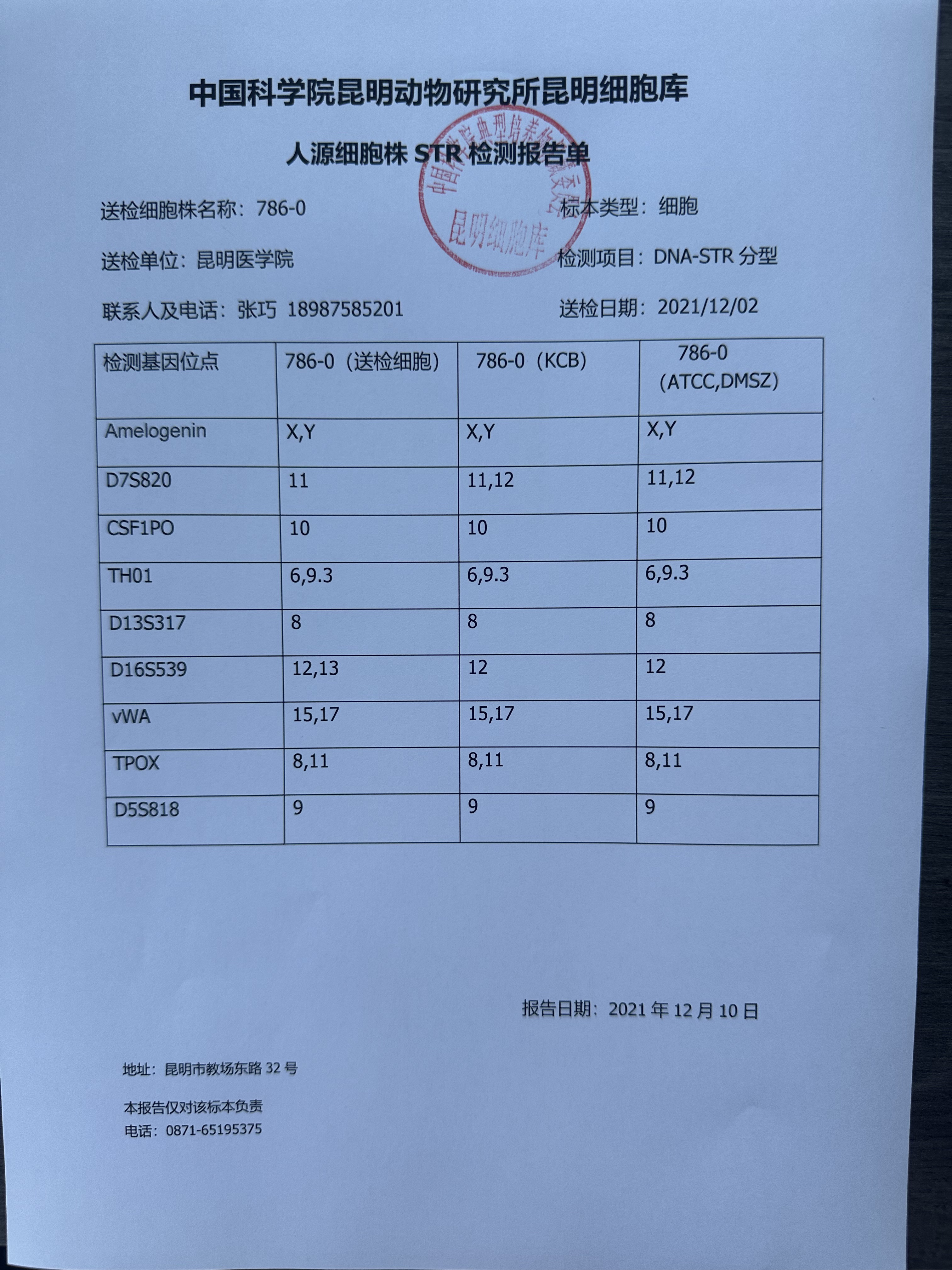

Supplement: Supplementary data 1 [file mmc1.zip › Supplementary data 1-STR identification certificates for cell lines/786-O.JPG]

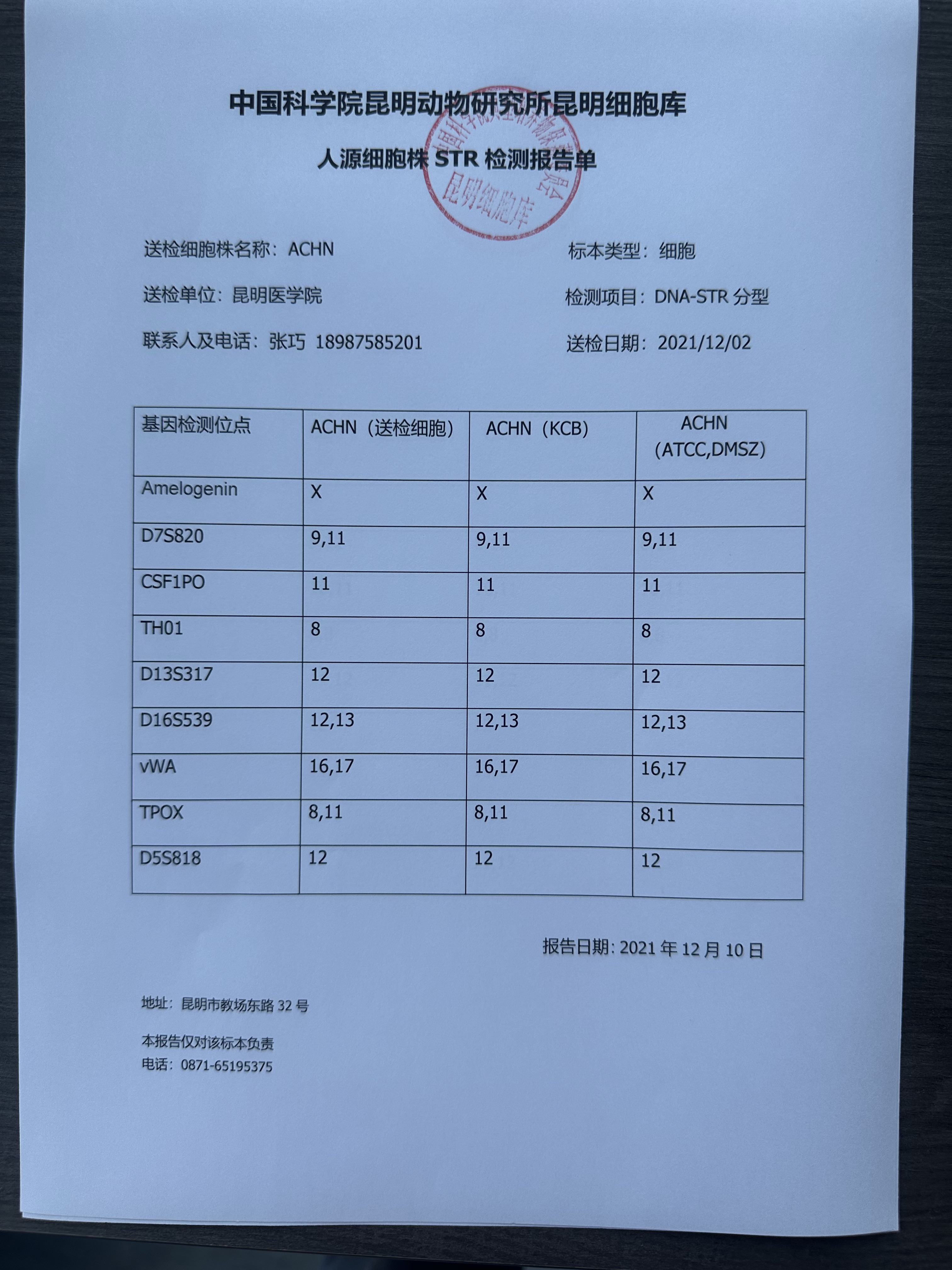

Supplement: Supplementary data 1 [file mmc1.zip › Supplementary data 1-STR identification certificates for cell lines/ACHN.JPG]

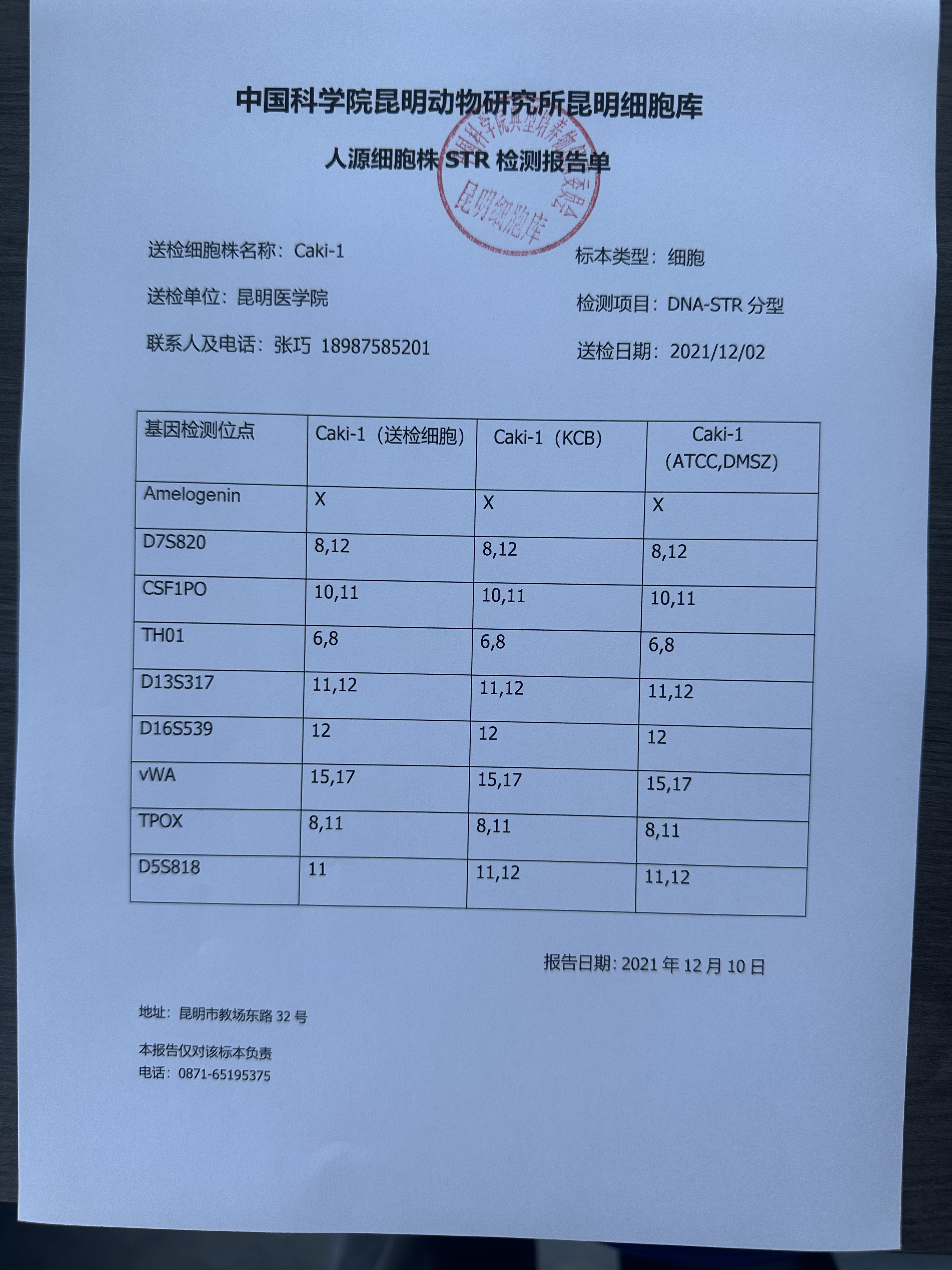

Supplement: Supplementary data 1 [file mmc1.zip › Supplementary data 1-STR identification certificates for cell lines/Caki-1.JPG]

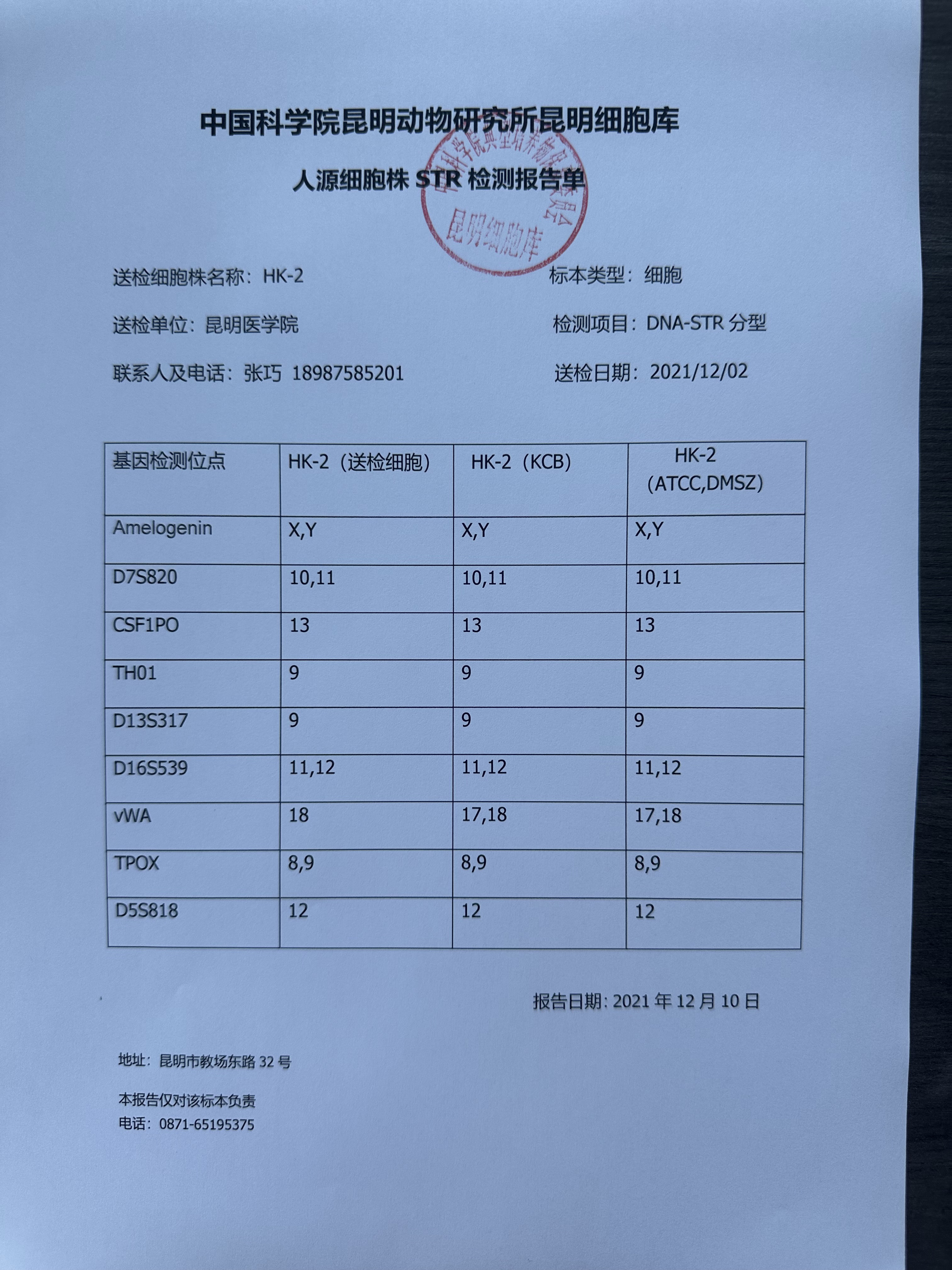

Supplement: Supplementary data 1 [file mmc1.zip › Supplementary data 1-STR identification certificates for cell lines/HK2.JPG]
