# Supplementary material for: A BRAF-activated noncoding RNA attenuates clear cell renal cell carcinoma via repression of glucose-6-phosphate dehydrogenase
Source: J Biol Chem. 2025 Jan 31;301(3):108247. doi: 10.1016/j.jbc.2025.108247 (PMC11889594; doi:10.1016/j.jbc.2025.108247)
Supplement: Supplementary data 3 [file mmc3.docx]

**
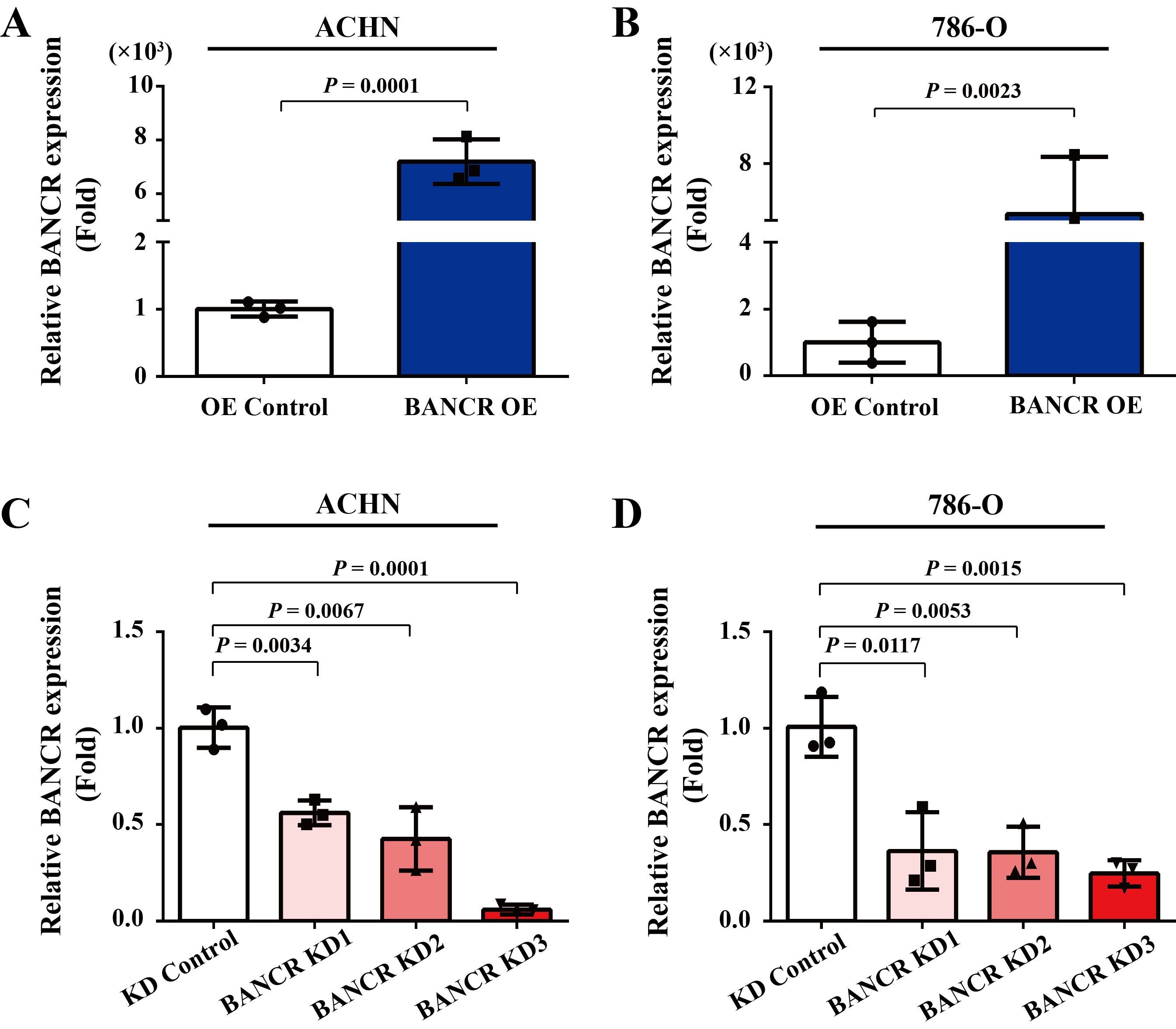
**

**Supplementary data 1 (Figure S1).**

**BANCR expression** **in stable BANCR overexpressing (A-B), BANCR knocked down (C-D) and relevant control cells were detected by Real-time RT-PCR analyses**. U6 was employed as a normalization control. Each analysis was performed at least three times. Data are shown as mean ± SD from three independent experiments, each performed in triplicate. unpaired Student’s *t*-test. OE, overexpression; KD, knockdown. These results demonstrated the successful establishment of both BANCR overexpressing and knocked down stable cell lines.


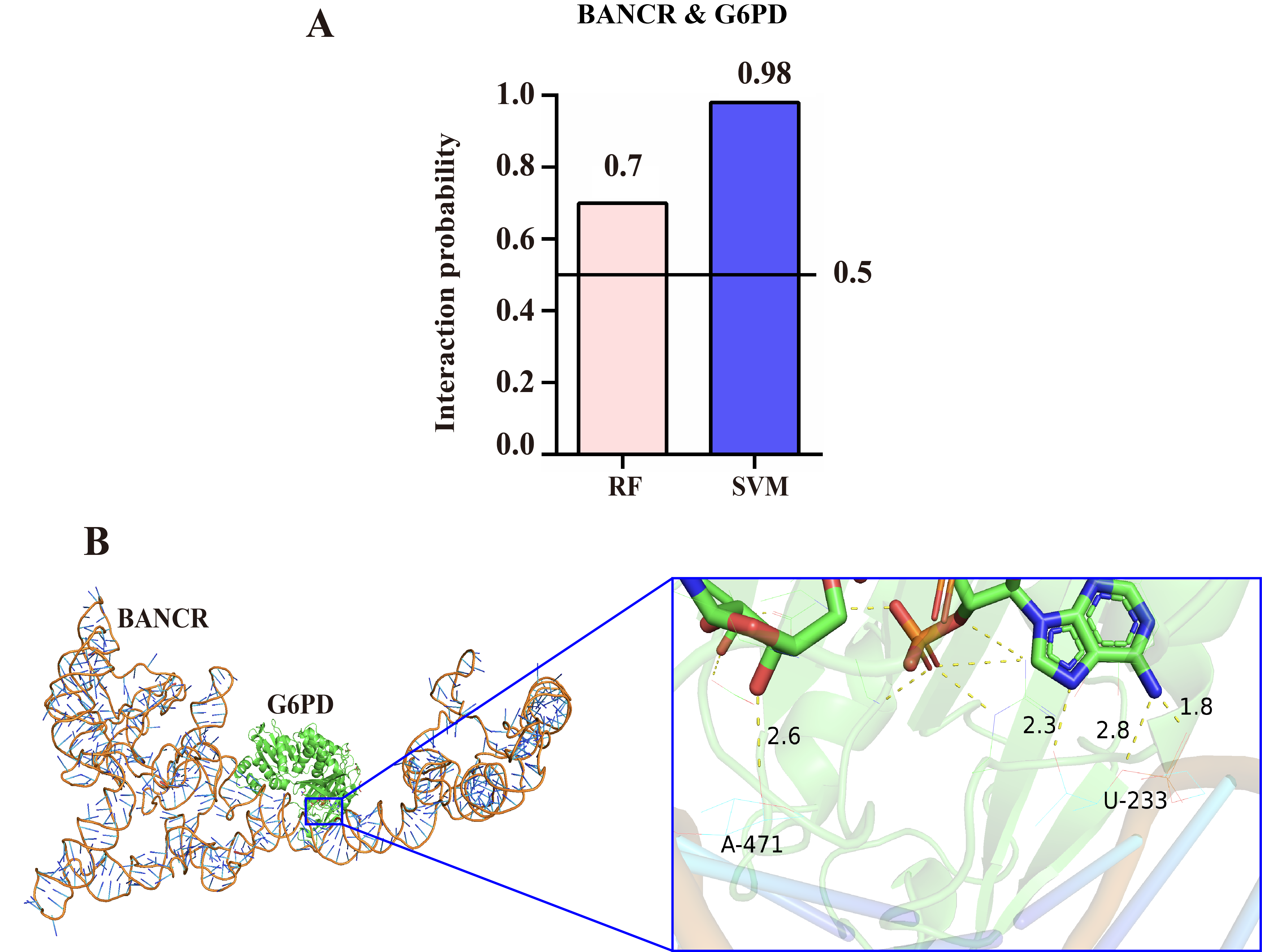


**Supplementary data 2 (Figure S2).**

**(A) The interaction between BANCR and G6PD was predicted on the RNA-Protein website** (http://pridb.gdcb.iastate.edu/RPISeq/index.html). The results demonstrated a significant likelihood of interaction between BANCR and G6PD, as indicated by RF=0.7 and SVW=0.98, both surpassing the threshold value of 0.5. **(B) Molecular docking between BANCER and G6PD were conducted**. The molecular structure of BANCR was obtained from HDOCK Server database (http://hdock.phys.hust.edu.cn), while the protein structure of G6PD was acquired from the PDB database (<https://www.rcsb.org>). The molecular docking was performed using AutoDock Vina, and the binding conformation with the lowest free binding energy was selected. Furthermore, to enhance prediction accuracy and identify the model with the highest confidence score, physical principles, coevolutionary residue coupling, and experimental contact information were incorporated. Finally, PyMOL 2.5.4 software (https://pymol.org/2/) was utilized for visual processing. The results demonstrated that G6PD formed four hydrogen bonds with U-233 and A-471 nucleobases of BANCER at distances of 1.8 Å, 2.8 Å, 2.3 Å, and 2.6 Å respectively. The *cat*RAPID software analysis revealed that the binding target G6PD most likely interacted with a base region of BANCR spanning from 276 to 327 nt. Interestingly, the molecular docking results for U233 and A471 precisely encompassed the predicted region of BANCR. Moreover, the docking analysis revealed a binding energy of -85234.90 for the interaction between BANCER and G6PD, suggesting a high likelihood of BANCR influencing the dimer formation and activity of G6PD.


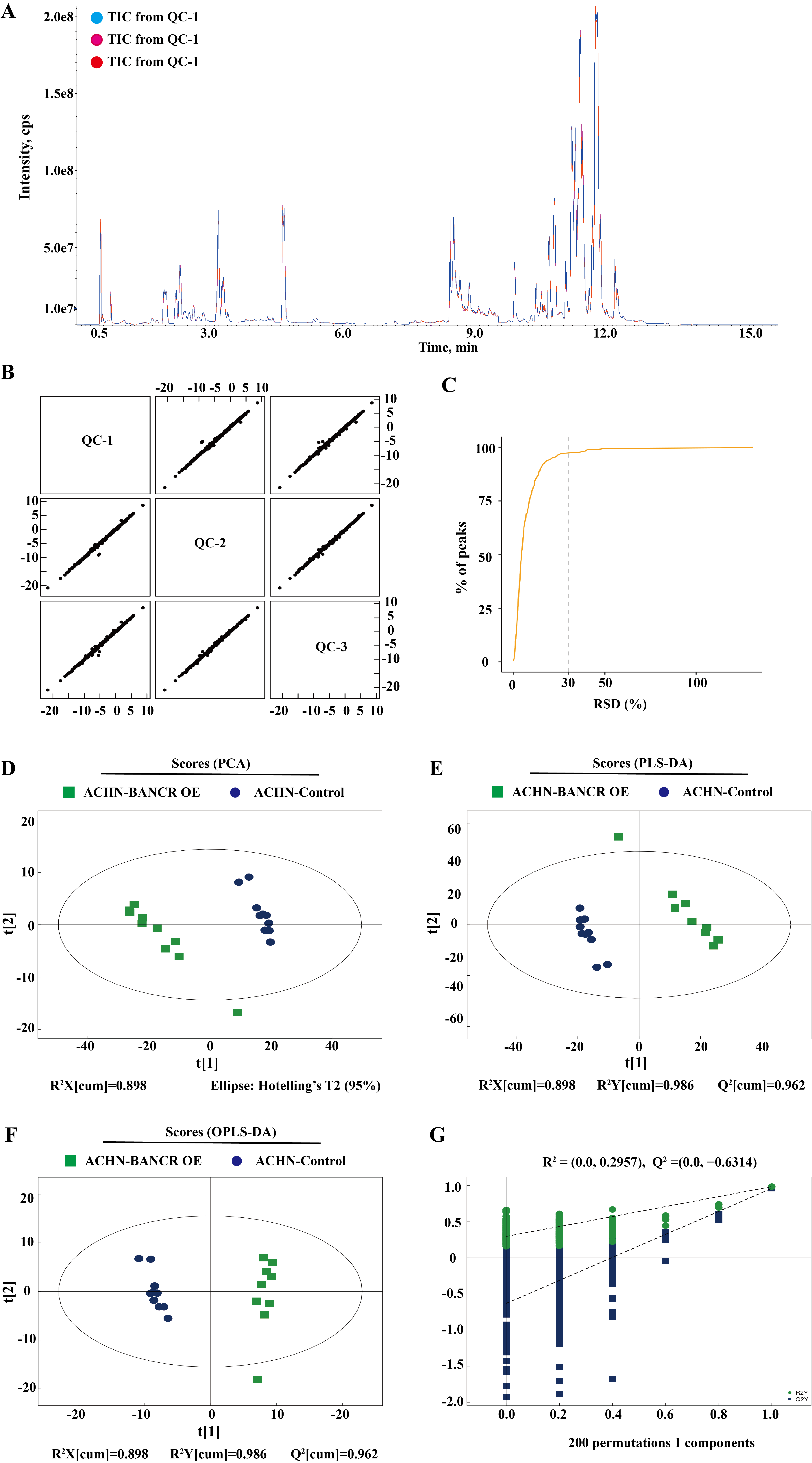


**Supplementary data 3 (Figure S3).**

**Targeted metabolomics analyses were conducted in ACHN-BANCR OE and relevant control cells** (n=10). **(A)** Total ion chromatogram of QC sample (C18 column). The results demonstrated a substantial overlap in both the response intensity and retention time of each chromatographic peak, indicating minimal variation attributed to instrumental error throughout the experimental process. **(B)** Correlation map of QC samples. The metabolite content in QC samples was normalized using the UV scale, and subsequently subjected to Pearson correlation analysis. The experimental findings demonstrated that the correlation coefficients among QC samples exceed 0.9, thereby indicating excellent experimental repeatability. **(C)** Relative standard deviation (RSD) distribution of QC samples, demonstrated that instrument fluctuations were within normal range, indicating high-quality data suitable for subsequent analyses. **(D)** Principal component analysis (PCA) showed high level of aggregation within the group and the distinct separation between the groups, suggesting that the model exhibited reliability, as well as significant disparities among the groups. **(E-F)** Partial least squares discrimination analysis (PLS-DA) and orthogonal partial least squares discrimination analysis (OPLS-DA) were shown. The model evaluation parameters obtained through 7-fold cross-validation, with Q^2^>0.5, indicated the stability and reliability of the model. **(G)** Permutation test of OPLS-DA demonstrated that R^2^ and Q^2^ of the random model exhibited a gradual decline as replacement retention decreased, indicating the absence of overfitting in the original model and highlighting its robustness.
